# Opening the tap: Increased riverine connectivity strengthens marine food web pathways

**DOI:** 10.1371/journal.pone.0217008

**Published:** 2019-05-23

**Authors:** Beatriz S. Dias, Michael G. Frisk, Adrian Jordaan

**Affiliations:** 1 Environmental Conservation Department, University of Massachusetts Amherst, Amherst, Massachusetts, United States of America; 2 CAPES Foundation, Ministry of Education of Brazil, Brasília-DF, Brazil; 3 School of Marine and Atmospheric Sciences, Stony Brook University, Stony Brook, New York, United States of America; Technical University of Denmark, DENMARK

## Abstract

Reduction of ecosystem connectivity has long-lasting impacts on food webs. Anadromous fish, which migrate from marine to freshwater ecosystems to complete reproduction, have seen their historically larger ecosystem role undercut by widespread riverine habitat fragmentation and other impacts mainly derived from anthropogenic sources. The result has been extensive extirpations and increased susceptibility to a suite of environmental factors that currently impede recovery. Under this present-day context of reduced productivity and connectivity, aggressive management actions and enforcement of catch limits including bycatch caps and complete moratoria on harvest have followed. What remains less understood are the implications of changes to food webs that co-occurred. What benefits restoration could provide in terms of ecosystem functioning in relation to economic costs associated with dam removal and remediation is unknown and can limit the scope and value of restoration activities. Here we employ, historical landscape-based biomass estimates of anadromous alosine for the first time in an ecosystem modeling of the Northeast US large marine ecosystem (LME), to evaluate the value of improving connectivity by measuring the increase in energy flow and population productivity. We compared a restored alosine model to a contemporary model, analyzing the impacts of the potential increase of connectivity between riverine and oceanic systems. There was the potential for a moderate biomass increase of piscivorous species with high economic value, including Atlantic cod, and for a major increase for species of conservation concern such as pelagic sharks, seabirds and marine mammals. Our study highlights the benefits of increased connectivity between freshwater and ocean ecosystems. We demonstrate the significant role anadromous forage fish could play in improving specific fisheries and overall ecosystem functioning, mainly through the diversification of species capable of transferring primary production to upper trophic levels, adding to benefits associated with their restoration.

## Introduction

Small pelagic finfish, characterized by extraordinary, yet highly variable abundance, are vital components of global food webs [1]. In the North Atlantic, these so-called forage fish make long migrations along the continental shelf in large schools of conspecifics (e.g., Atlantic menhaden [*Brevoortia tyrannus*], [2]) or among mixed species (e.g., Atlantic herring [*Clupea harengus*], mackerel [*Scomber scombrus*] and river herring [3]). They feed almost exclusively on planktivorous organisms as juveniles, and most add small invertebrates and fishes to their diets as adults. At all life stages, forage fish transfer primary production to higher trophic levels as they are consumed by diverse marine predators, including bony fish, elasmobranchs, marine mammals, and seabirds [4].

Ecosystem connectivity, the movement of energy, inert material, nutrients and organisms across physical or biological system boundaries, enhances the function and health of aquatic ecosystems [5,6]. Forage fish add substantially to ecosystem connectivity by translocating nutrients along migratory highways in their seasonal processions from spawning grounds to feeding grounds. Occupying distinct habitats as temporary inhabitants of coastal and marine ecosystems, pulses of prey species enrich successive food bases along the way [7], simultaneously providing trophic and geographic connectivity, and supporting vital coastal and offshore fisheries.

Historical records and recent research correlate the seasonal occurrence of forage fish species to the movements and habitat preferences of cod and other groundfish [8,9]. It should not be surprising, then, that loss of forage species is associated with marine ecosystem decline. Deficient quantity and quality of the forage base have been linked to apex predator’s poor physical condition, low productivity, and the failure of population recovery after depletion events [10,11]. Along with global warming, spatiotemporal mismatch with lipid-rich prey may reduce even more the productivity in highly valuable fished populations, such as the Gulf of Maine’s Atlantic cod stocks (*Gadus morhua*), exacerbating their decline, or impairing their recovery [12]. The recent recovery of capelin (*Mallotus villosus*), a lipid-rich forage species, spurred growth in Newfoundland’s cod stocks, depressed since the mid-1990s [13]. As warming waters continue to shift the spatial range and timing of fish migrations, mismatches caused by reduced predator and prey overlap becomes more frequent [14].

Whereas questions remain about the importance of single predator-prey linkages in driving productivity across larger ecosystems [15], complex life histories likely contribute consistency to predator-prey relationships [16,17]. For instance, capelin have two spawning modes, both of which contribute to stock productivity [18,19]. Forage species that spawn in freshwater or brackish estuaries and marshes only enter the marine food web after their eggs and larvae develop into juvenile fish, and thus they may play complementary, but different ecosystem roles compared to marine spawners like Atlantic herring.

**Fig 1:**
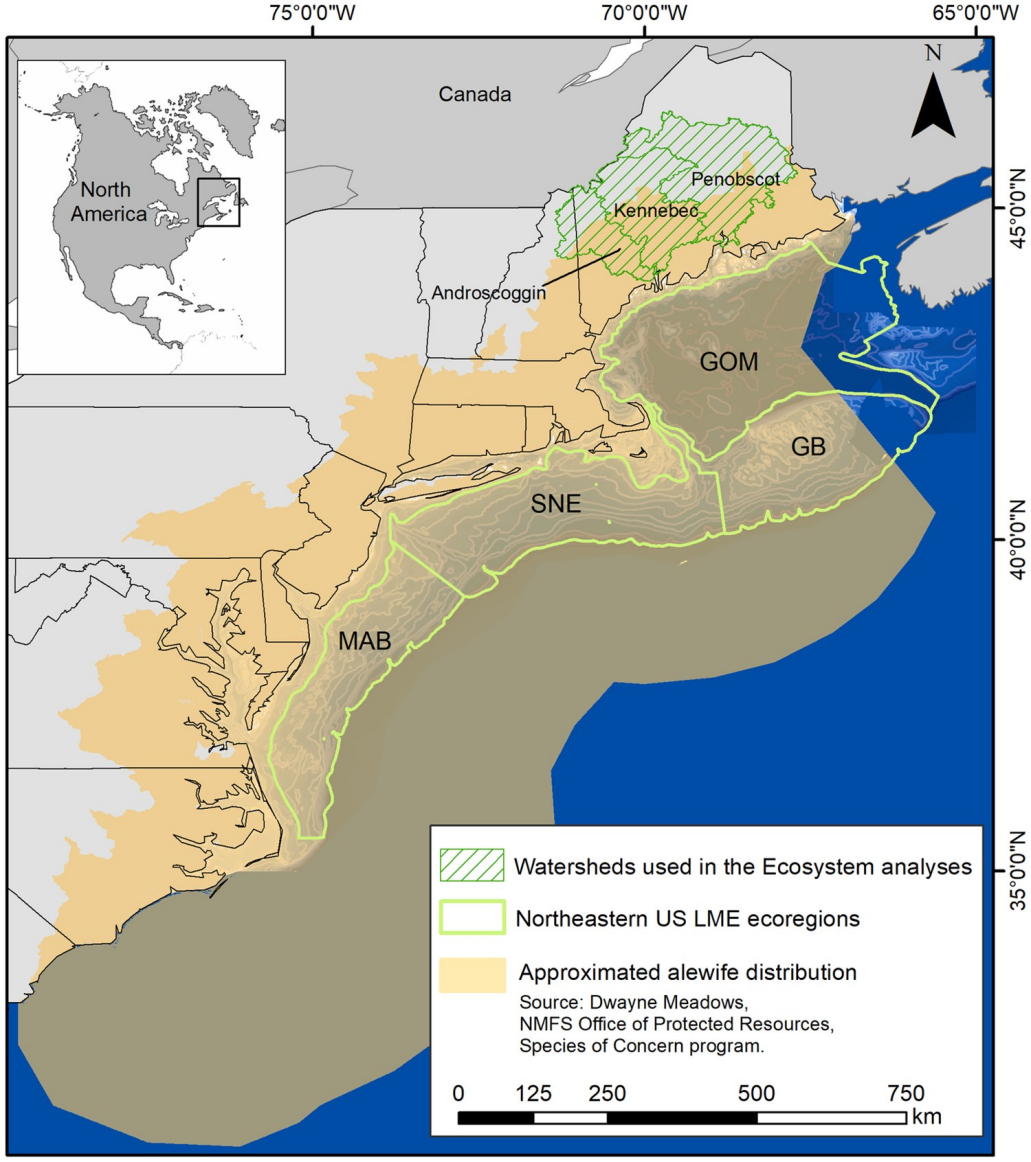
Map of the study area and sub-regions included in both models. This map shows the bathymetric profile of the coastal region, and NEUS LME ecoregions: The Gulf of Maine (GOM), Georges Bank (GB), Southern New England (SNE), and Middle Atlantic Bight (MAB). The limits of the tan region also represent the US Exclusive Economic Zone (EEZ).

River herring, anadromous alosines including alewife (*Alosa pseudoharengus*) and blueback herring (*Alosa aestivalis*), are coastal forage species that spend most of their lives at sea, where schools of adults often merge with larger schools of mature Atlantic herring and mackerel [3,20,21]. Every year, however, most return to fresh water to spawn in natal grounds [22]. Extreme abundance of these fish in the Northeast US Large Marine Ecosystem (LME) (Fig 1) and their annual transition between fresh- and saltwater, ensured a strong flow of energy between marine and upland ecosystems [23] and abundant forage for predators, particularly where rivers join the sea. However, river herring stocks throughout the LME were depleted as dams impeded or blocked upwards of 95% of freshwater spawning habitat compared to pre-colonial conditions [23,24]. Linkages between marine and freshwater systems unraveled [25] as these key prey species became functionally extinct throughout most of their range.

**Fig 2:**
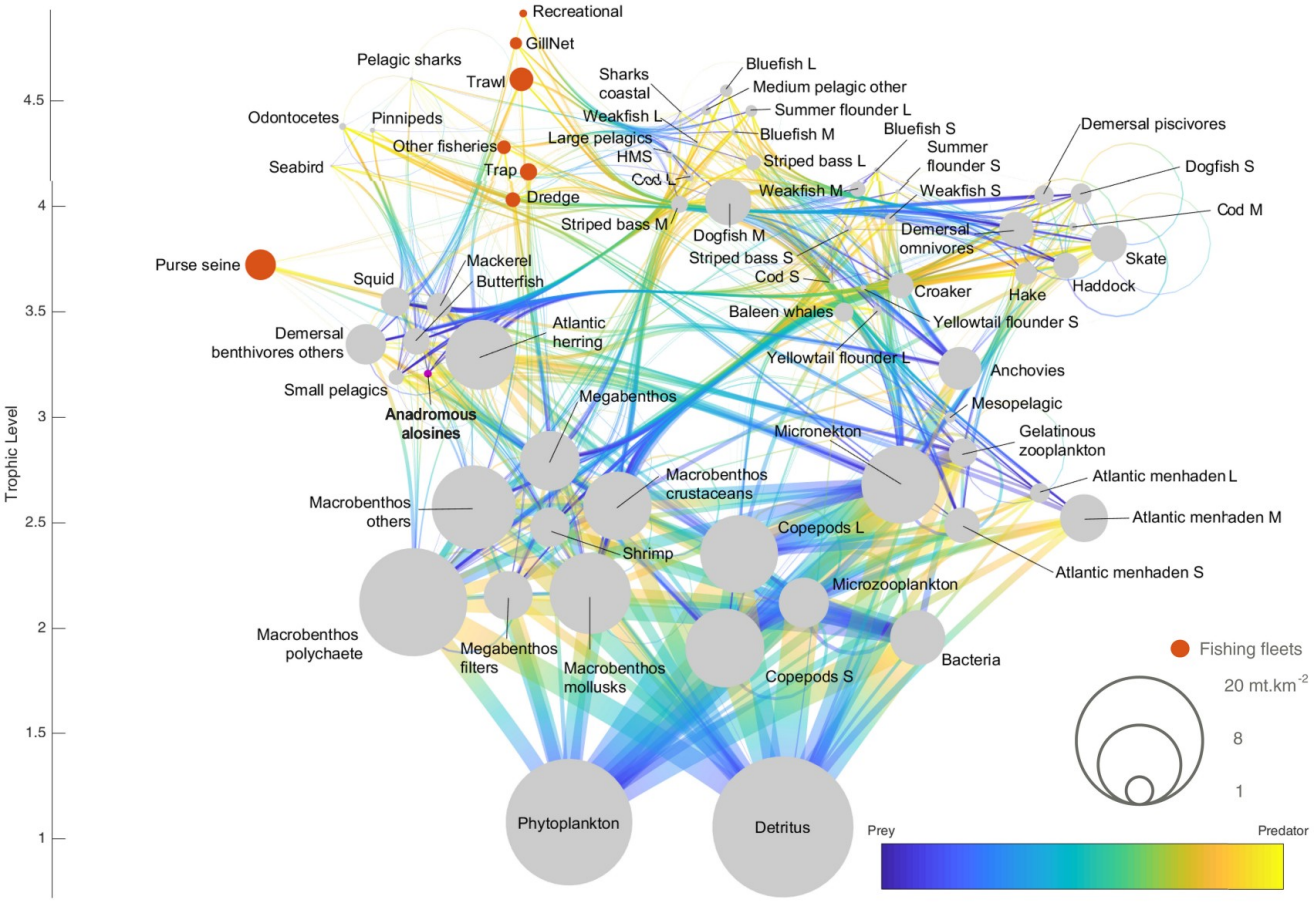
Flow diagram of the Contemporary Alosine Biomass model. The color gradient represents the direction of flow; different life stages are represented by small (S), medium (M) and Large (L). Functional groups are ordered by trophic level. Grey bubbles represent all functional groups, the pink bubble in bold letters represents anadromous alosine, and orange bubbles represent fishing fleets.

Current interest in the status of alewives and the success of dam removal and improved fish passage in increasing alewife abundance, particularly in Maine [26,27], encouraged us to test, via ecosystem modeling, the impacts of increasing anadromous forage fish populations on marine food webs. First, we estimated potential alewife production in three Maine watersheds (Androscoggin, Kennebec, and Penobscot) based on the spawning habitat potentially available to them. Then, we employed that estimate in an Ecopath with Ecosim model framework to assess how significantly increasing forage might impact predators in the Northeast US (NEUS) LME (Fig 1). We built two EwE models for comparison. The Contemporary Alosine Biomass (CAB) model reflects actual ecosystem conditions in the year 2000 (Fig 2). The Restored Alosine Biomass (RAB) model incorporates estimated alewife production on the three watersheds before 1600, prior to dam construction (Fig 1). Because alewives spawn far inland and are sensitive to river fragmentation and other environmental alterations [28], the RAB scenario assumes that adult biomass scales linearly with access to spawning habitat. Specifically, the objectives were: 1) to quantify estimates of biomass change for managed species targeted by fisheries or are species of concern; 2) to quantify changes in biomass flows from middle to upper trophic levels; 3) to provide context for the role of anadromous forage fish in the NEUS LME, the historical loss, and the impacts of river restoration on marine ecosystems.

Rather than match the spatial extent of our models to the spatial scale of our historical estimates (the Gulf of Maine), we chose instead to model the entire NEUS LME. The approach conforms to modern stock assessment methods and management where population assessments are generally conducted over the whole range of a species or stock (within national boundaries). Alewife stocks extend from Labrador to North Carolina [29], and Gulf of Maine populations are likely to occupy a broader region throughout the NEUS LME during the three to four years of full marine occupancy. Restoration goals were established based on data from the second half of the 20^th^ century [30], as they were intended for other managed marine species within the LME. Setting restoration targets to recent baselines neglect both the historical productivity of individual species and the system productivity derived from trophic integrity and connectivity and in this case a long history of habitat loss undermining these key aspects.

Evolution from single species to ecosystem-based management (EBM) requires understanding trophic interactions and anthropogenic disturbances across variable temporal and spatial scales [31]. Here, we employ a novel deployment of EwE to explore the value of increasing forage species abundance, including consequences on predators, improvements to environmental health, delivery of ecosystem services, and human well-being.

## Materials and methods

### Species of interest

To assess the impacts of a potential increase in forage fish biomass on the marine environment, we focused on alewife (*A*. *pseudoharengus*). Alewife is the flagship species within the anadromous alosine group for several reasons. Due to spawning habitat preferences, they are most vulnerable to changes in river connectivity, but they are also good indicators of the health of other anadromous species that spawn in rivers and the upper bounds of estuaries [23,32]. Moreover, they have the highest potential for population restoration among anadromous species [33], and have been the most responsive to increases in spawning habitat after dam removal. Unlike menhaden or Atlantic herring, which support managed fisheries and are considered to be at adequate population levels, alewife is a candidate for protection under the US Endangered Species Act [34], and catching the fish is banned throughout much of their US range, except for the State of Maine. Concerted state and federal efforts are underway to restore access to spawning habitat along alewife’s range, including the three major watersheds considered here.

Our study is based on previous work by Hall *et al*. [28] and Mattocks *et al*. [23], where they focused on alewife historic spawning habitat (lakes and ponds) and productivity rates for the species, however they did not provide comparable estimates for blueback herring and American shad, therefore we exclude the biomass reconstruction for these species under the anadromous alosines group. Since 2013, NOAA’s National Marine Fisheries Service has been committed to working with the Atlantic States Marine Fisheries Commission to fill data gaps regarding the biology of alewives and blueback herring, yet aspects of blueback herring ecology and biology remain unknown. We acknowledge that modeling a single species in the anadromous alosine group is underestimating the full benefits of fish passage. Nevertheless, this underestimation helps ensure that our results are conservative in scope. Our analysis was motivated to understand the consequence of increasing alewife biomass in the NEUS LME.

### The ecosystem modeling approach

We built two ecosystem models using the Ecopath with Ecosim framework (EwE 6.0, [35]) to assess and quantify ecosystem-level biomass changes resulting from alosine biomass restoration. Originally developed to address questions regarding ecosystem structure, dynamics and external drivers, such as fishery harvest [36–38], the mass-balance ecotrophic model represents the ecosystem as functional groups or nodes (different species, ontogenetic phases or groups with the same ecological importance) connected by trophic relationships. Our model, based on Ecopath, the core routine of EwE, provides a static snapshot of a “closed” ecosystem, where no imports with adjacent ecosystems were considered [39,40]. The links between the nodes represent trophic interactions estimated from published diet studies. Thus, diet composition determines energy and matter flow throughout the system in each time period. Ecopath’s main equation takes the following form:

$$P_{i}=B_{i}∙{M2}_{i}+Y_{i}+E_{i}+{BA}_{i}+P_{i}∙(1-{EE}_{i})$$ (1)

where, for a given group (*i*), *P*_*i*_ is production, *B*_*i*_ is biomass, *M2*_i_ is the total predation mortality rate for group (*i*), *Y*_*i*_ is the total fishery catch rate, *E*_*i*_ is net-migration rate, *BA*_*i*_ is biomass accumulation rate for (*i*), *EE*_*i*_ is ecotrophic efficiency (the proportion of the production used in the system), and *P*_*i*_.*(1-EE*_*i*_*)* represents the rate of other sources of mortality for (*i*) [41].

The following equation expresses the relationship between predator and prey:

$$B_{i}∙{M2}_{i}=\sum_{j=1}^{n}{(B_{j}∙(Q/B)}_{j}∙{DC}_{ji})$$ (2)

Where the biomass times the predation mortality of prey (*i*) equals the sum across all the predators (*j*) of the predator biomass *B*_*j*_ times the consumption per unit biomass of (*j*) *(Q/B)*_*j*_ times the fraction of prey group (*i*) in the diet of predator group (*j*) *DC*_*ji*_ [42]. The Ecopath modeling framework assumes that consumption equals production plus respiration and unassimilated food. This equation is the representation of mass-balanced hypothesis.

These two main equations yield the following full linear equation for a given period. Eq 1 can be rewritten as:

$$B_{i}∙{\left(P/B\right)}_{i}{-\sum_{j=i}^{n}B_{j}∙(Q/B)}_{j}∙{DC}_{ji}–({P/B)}_{i}∙B_{i}∙(1-{EE}_{i})-Y_{i}-E_{i}-{BA}_{i}=0$$ (3)

or

$$B_{i}∙{\left(P/B\right)}_{i}{∙EE}_{i}-{\sum_{j=i}^{n}B_{j}∙(Q/B)}_{j}∙{DC}_{ji}-Y_{i}-E_{i}-{BA}_{i}=0$$ (4)

where (*P/B)*_*i*_ is the production of the functional group (*i*) per unit of biomass [35, 41–43].

The ecotrophic efficiency term *EE*_*i*_ is solved by Eq 5:

$${EE}_{i}=(Y_{i}+E_{i}+{BA}_{i}+{M2}_{i}∙B_{i})/P_{i}$$ (5)

The ecotrophic efficiency varies between 0 and 1 and can be expected to approach 1 for groups with high predation and exploitation pressures; this value is used here for tuning the model. For groups where EE value is superior to 1, the remainder of parameters should be tuned during the model parametrization, also known as the balancing process [41]. EwE’s multistanza function accounts for the ontogenetic differences between life stages. We first built a fully balanced model using the multistanza approach. However, we forewent utilizing this feature. Instead, we conserved ontogenetic groups as different nodes to simplify comparing changes in biomass in the two models. To calculate production for each age node, we used the following trophic and growth-based production model [44],

$$P/B=2.56\tau^{-.78}K^{.7}e^{\left(.02*\theta \right)}$$ (6)

Where *Ʈ* is the trophic level (calculated by the first model using the multistanza approach from diet data information), *K* is the von Bertalanffy growth parameter of each species, and *θ* is water temperature, which we estimated using the mean temperature from each species’ spatial range in the NEUS LME (Table E in S1 File). As described by the equations above, the Ecopath’s main input parameters are B, P/B, Q/B, EE and diet regimes. Not all the parameters used to construct an Ecopath model need to be entered; therefore missing parameters will be estimated by the model using the balanced sets of equations.

### Functional groups

The models were based on four EwE Models built for the Energy Modeling and Analysis eXercise (EMAX) project [45,46] with the NOAA Northeast Fisheries Science Center (NEFSC) data. The EMAX models presented an average of 36 functional groups per region, with low taxonomic resolution. To create our baseline model (CAB), we averaged EMAX inputs and expanded the functional groups to achieve higher taxonomic resolution. We separated key ecological or economically important species into different ontogenetic groups and resulted in a total of 59 functional groups (Table 1).

**Table 1:** Functional groups input parameters sources for the Contemporary Alosine Biomass (CAB) model for the NEUS LME. Inputs parameters are Biomass (B), the production-biomass ratio (P/B) and the consumption-biomass ratio (Q/B), output parameters calculated by EwE are Trophic level (TL), Ecotrophic Efficiency (EE) and the production-consumption ratio (P/Q), signaled in bold. Input data documentation is found in the S1 File.

Node	Group name	TL	B (t.km^-2^)	P/B (y^-1^)	Q/B (y^-1^)	EE	P/Q (y^-1^)
1	Phytoplankton	**1.00**	20.13	180.69		**0.58**	
2	Bacteria	**2.00**	3.83	91.25	**182.50**	**0.88**	0.50
3	Microzooplankton	**2.22**	3.16	72.00	242.42	**0.54**	**0.30**
4	Copepods S	**2.10**	7.81	42.58	127.75	**0.98**	**0.33**
5	Copepods L	**2.23**	7.63	48.52	109.50	**0.90**	**0.44**
6	Gelatinous Zooplankton	**2.93**	1.01	37.97	145.33	**0.67**	**0.26**
7	Micronekton	**2.73**	7.65	14.25	85.50	**0.79**	**0.17**
8	Macrobenthos polychaete	**2.34**	14.68	2.51	17.50	**0.98**	**0.14**
9	Macrobenthos crustaceans	**2.62**	5.90	3.06	21.00	**0.79**	**0.15**
10	Macrobenthos mollusks	**2.28**	8.34	2.04	13.95	**0.94**	**0.15**
11	Macrobenthos others	**2.48**	8.90	2.02	16.06	**0.95**	**0.13**
12	Megabenthos filters	**2.11**	3.00	3.94	16.51	**0.20**	**0.24**
13	Megabenthos others	**2.97**	4.50	1.90	9.53	**0.63**	**0.20**
14	Shrimp	**2.80**	1.96	1.00	5.00	**0.50**	**0.20**
15	Mesopelagic	**3.27**	0.15	0.65	1.83	**0.75**	**0.36**
16	Atlantic herring	**3.51**	6.20	0.62	4.59	**0.61**	**0.14**
17	Anadromous alosines	**3.40**	0.08	1.30	9.40	**0.90**	**0.14**
18	Atlantic menhaden S	**2.50**	1.58	1.50	15.86	**0.54**	**0.09**
19	Atlantic menhaden M	**2.64**	2.88	0.93	7.01	**0.50**	**0.13**
20	Atlantic menhaden L	**2.78**	0.49	0.90	4.38	**0.86**	**0.21**
21	Anchovies	**3.70**	2.32	3.00	10.90	**0.76**	**0.28**
22	Mackerel	**3.83**	0.77	0.39	1.98	**1.00**	**0.20**
23	Squid	**3.71**	1.06	0.98	2.70	**0.83**	**0.36**
24	Butterfish	**3.59**	0.90	1.27	1.98	**0.42**	**0.64**
25	Small pelagics	**3.37**	0.29	0.97	4.00	**0.89**	**0.24**
26	Bluefish S	**4.36**	0.05	0.51	18.11	**0.94**	**0.03**
27	Bluefish M	**4.44**	0.06	0.51	3.53	**0.67**	**0.14**
28	Bluefish L	**4.64**	0.19	0.49	1.93	**0.14**	**0.25**
29	Striped bass S	**3.99**	0.07	0.25	23.27	**0.78**	**0.01**
30	Striped bass M	**4.05**	0.37	0.25	6.35	**0.19**	**0.04**
31	Striped bass L	**4.23**	0.29	0.24	3.19	**0.20**	**0.08**
32	Weakfish S	**4.07**	0.16	0.45	13.52	**0.92**	**0.03**
33	Weakfish M	**4.28**	0.30	0.43	4.22	**0.09**	**0.10**
34	Weakfish L	**4.35**	0.04	0.42	2.45	**0.48**	**0.17**
35	Dogfish S	**4.06**	0.47	0.25	1.47	**0.79**	**0.17**
36	Dogfish L	**4.09**	2.70	0.24	0.61	**0.07**	**0.40**
37	Atlantic cod S	**3.63**	0.03	0.48	6.91	**0.81**	**0.07**
38	Atlantic cod M	**3.92**	0.08	0.46	3.49	**0.96**	**0.13**
39	Atlantic cod L	**4.19**	0.08	0.43	2.26	**0.96**	**0.19**
40	Haddock	**3.69**	0.60	0.45	3.00	**0.45**	**0.15**
41	Hake	**3.81**	0.83	1.12	3.85	**0.64**	**0.29**
42	Croaker	**3.59**	0.82	0.45	0.91	**0.33**	**0.50**
43	Yellowtail flounder S	**3.60**	0.04	1.07	4.41	**0.17**	**0.24**
44	Yellowtail flounder L	**3.49**	0.11	1.10	2.90	**0.46**	**0.38**
45	Summer flounder S	**4.25**	0.03	0.56	4.41	**0.64**	**0.13**
46	Summer flounder L	**4.54**	0.18	0.53	2.90	**0.48**	**0.18**
47	Skate	**3.83**	1.66	0.45	2.40	**0.29**	**0.19**
48	Demersal benthivores	**3.62**	2.05	0.45	0.91	**0.96**	**0.50**
49	Demersal piscivores	**4.13**	0.55	0.55	1.21	**0.95**	**0.45**
50	Demersal omnivores	**3.96**	1.50	0.45	0.81	**0.87**	**0.55**
51	Medium pelagic	**4.54**	0.12	0.45	1.84	**0.06**	**0.24**
52	Coastal sharks	**4.53**	0.02	0.20	1.25	**0.95**	**0.16**
53	Pelagic sharks	**4.59**	0.02	0.11	0.69	**0.32**	**0.16**
54	Large pelagics (HMS)	**4.31**	0.07	0.58	6.79	**0.83**	**0.09**
55	Pinnipeds	**4.49**	0.04	0.08	5.50	**0.25**	**0.01**
56	Baleen whales	**3.47**	0.46	0.04	3.22	**0.03**	**0.01**
57	Odontocetes	**4.49**	0.06	0.04	14.30	**0.60**	**0.00**
58	Seabirds	**4.27**	0.01	0.28	9.32	**0.42**	**0.03**
59	Detritus	**1.00**	52.61			**0.51**	

### Model scenarios

We developed EwE models of the Northeast US LME to explore the potential marine ecosystem effects of increasing anadromous alosine biomass by reestablishing full river to ocean connectivity on the three Northern New England Watersheds: the Androscoggin, Kennebec, and Penobscot river systems (total of 1.280 km^2^ of lake/pond area). Both the Contemporary Alosine Biomass Model (CAB) and the Restored Alosine Biomass Model (RAB) were built with the same spatial structure, encompassing the full range of alewife (Fig 1) in the NEUS LME: the Gulf of Maine, Georges Bank, Southern New England, and Middle Atlantic Bight (246,662 km^2^). However, RAB assumed restored alewife biomass based on historical landscape estimates in Mattocks *et al*. [23], which resulted in a biomass input of 137,637 mt for the anadromous alosine group. The CAB model anadromous alosine group biomass estimate used was 0.08 t.km^-2^, while RAB estimate was 0.63 t.km^-2^. Thus, it reflects the potential habitat expansion on these Northern New England Watersheds (Fig 1).

### Timeframe analysis

The models use the year block 2000 as the reference point for biomass, consumption, production, diets, mortality and fishing mortality. This year block, comprising the years 1996 to 2000, was chosen for use in the four EMAX Models due to the amount of available data.

### Data sources

To build our baseline model of current conditions (CAB), we used sources including EMAX Model raw input data, EMAX model balanced results, NEFSC trawl surveys, stock assessments, and scientific literature. Our initial Ecopath parameter inputs (Biomass, Production, Consumption, and Diets) came from weighted averages of the combined regions of the EMAX models. Using these weighted averages, we calculated total biomass estimates for the Northeast US LME area. The same process was applied to calculating production. Since consumption was based on the amount of food ingested by a population relative to its biomass (in a given year, [47]), the consumption biomass (*Q/B*) ratio was consistent among all EMAX regions. For diet data, we used raw inputs from EMAX and from the Virginia Institute of Marine Science Fish Food Habits database, which were modified during the balancing process (S1 File). Pre-balancing was performed with PREBAL pre-balancing methodology [48] (Fig A in S1 File), and balancing followed the guidelines in Heymans et al. [49]. Once the CAB model was balanced, we generated the flow diagram (Fig 2) using the ecopath_matlab toolbox [50].

The model representing conditions without dams (RAB) was built in two steps. First, we applied alewife historical productivity data based on landscape estimates that assumed full river to ocean connectivity for the Northern New England Watersheds. These estimates were derived from Mattocks *et al*. [23] and Hall *et al*. [28], who calculated declining alewife production in lakes and ponds throughout New England from the year that dams began to obstruct the rivers. The total lake/pond area (km^2^) and the total length of pre-dammed rivers provide the total historical alewife spawning habitat (Fig 1). Both studies based habitat loss on species-specific spawning habitat preferences. Since alewife prefers spawning in still water, we calculated total un-dammed lake and pond area in square kilometers (km^2^).

For the second step of the RAB model, we defined small pelagics and forage fish, and analyzed diet information to identify all functional groups that presented trophic interactions with anadromous alosine and other forage fish groups. We used the ecotrophic efficiencies from the CAB model to calculate new biomass estimates for the key functional groups that incorporate the additional historical alewife biomass in the anadromous alosine group (Alewife *A*. *pseudoharengus*, blueback herring *A*. *aestivalis*, and American shad *A*. *sapidissima*).

We analyzed the impacts on the marine environment of increasing forage fish biomass, in the form of alewives (*Alosa pseudoharengus*) within the alosine functional group, by first calculating lost alosine productivity due to river impediment. Using methods in Mattocks *et al*.[23] and Hall *et al*. [28], we estimated the potential young of the year (YoY) productivity. The average YoY alewife density in 18 ponds, determined by field surveys, was applied to the total accessible pond and lake area for the three Northern New England watersheds,

$$N_{t}=A∙D_{Y}$$ (7)

where *N*_*t*_ is the potential number of alewife YoY produced before emigration to the marine habitat, *D*_*Y*_ is the YoY density of sampled lacustrine habitat (number of fish ∙ km^-2^), and *A* is the total pond and lake area within watersheds.

An exponential model of population growth was used to estimate subsequent alewife year classes,

$$N_{t+1}=N_{t}e^{-Z}$$ (8)

to predict the abundance of alewives at years two, three and four. *N* is the number of fish at time *t*, and *Z* is the annual instantaneous (total) mortality rate of 0.8 [25]. After hatching, alewives spend part of their first summer in their natal freshwater nursery habitat, and migrate to coastal waters through the summer and fall of their first year [51,52]. Thus, we could estimate total biomass using the resulting abundance and mean biomass at age (Tables J and K in S1 File). For fish in the 4+ age class, we used the mean weight shown in Hall *et al*. [28]. For other age classes, we calculated weight using the fork length-weight (in grams) relationship [53],

$$W=2.42∙10^{-6}∙{FL}^{3.34}$$ (9)

where *FL* (in mm) is fork length. FL data came from the Maryland Department of Natural Resources (MDNR) in a long-term dataset collected since 1989.

Both models had 59 functional groups (S1 File) determined by ecological role and trophic level. The Contemporary Alosine Biomass (CAB) model used biomass (B), consumption (Q/B), production (P/B), and diets (DC) from stock assessments, NEFWS trawl survey, and fishbase.org. The model estimated Ecotrophic efficiency (EE). As input, the Restored Alosine Biomass (RAB) model employed the potential alewife biomass of the Northern New England Watersheds fully connected to the ocean. Using EE, P/B, Q/B as input parameters allowed the model to calculate the biomass of various species of economic and conservation interest, except for apex predator functional groups, for which EE approximated zero (Table 2) [49]. We verified our estimates by running the RAB model biomass outputs and alosine restored biomass as our input parameters to confirm that we obtained the same EE for both models. We assumed that the EE parameter for anadromous alosine would remain high after biomass reconstruction for alewife, as they are a forage fish. During the balancing process for RAB model, we modified the diets to account for the increase of anadromous alosine biomass. We also increased the biomass for macrobenthos polychaetes, crustaceans and others to accommodate the increase in biomass of their predators (S1 File).

**Table 2:** Functional groups input parameters sources for the Restored Alosine Biomass (RAB) model for the NEUS LME. Inputs parameters are the production-biomass ratio (P/B), the consumption-biomass ratio (Q/B), and Ecotrophic Efficiency (EE) from CAB model. Output parameters calculated by EwE are Trophic level (TL), Biomass (B) and the consumption-production ratio (P/Q), signaled in bold.

Node	Group name	TL	B (t.km^-2^)	P/B (y^-1^)	Q/B (y^-1^)	EE	P/Q (y^-1^)
1	Phytoplankton	**1.00**	20.13	180.69		**0.58**	
2	Bacteria	**2.00**	3.83	91.25	**182.5**	**0.90**	0.50
3	Microzooplankton	**2.22**	3.16	72.00	242.42	**0.55**	**0.30**
4	Copepods S	**2.10**	7.81	42.58	127.75	**0.82**	**0.33**
5	Copepods L	**2.23**	7.63	48.52	109.50	**0.92**	**0.44**
6	Gelatinous Zooplankton	**2.93**	1.01	37.97	145.33	**0.69**	**0.26**
7	Micronekton	**2.62**	7.65	14.25	85.50	**0.85**	**0.17**
8	Macrobenthos polychaete	**2.33**	14.92	2.51	17.50	**0.93**	**0.14**
9	Macrobenthos crustaceans	**2.55**	6.30	3.06	21.00	**1.00**	**0.15**
10	Macrobenthos mollusks	**2.28**	8.34	2.04	13.95	**0.84**	**0.15**
11	Macrobenthos others	**2.47**	9.39	2.02	16.06	**0.79**	**0.13**
12	Megabenthos filters	**2.11**	3.00	3.94	16.51	**0.23**	**0.24**
13	Megabenthos others	**2.87**	4.50	1.90	9.53	**0.80**	**0.20**
14	Shrimp	**2.78**	**3.02**	1.00	5.00	0.50	**0.20**
15	Mesopelagic	**3.25**	**0.27**	0.65	1.83	0.75	**0.36**
16	Atlantic herring	**3.44**	**10.41**	0.62	4.59	0.61	**0.14**
17	Anadromous alosines	**3.36**	0.63	1.30	9.40	0.90	**0.14**
18	Atlantic menhaden S	**2.50**	**2.02**	1.50	15.86	0.54	**0.09**
19	Atlantic menhaden M	**2.64**	**3.39**	0.93	7.01	0.50	**0.13**
20	Atlantic menhaden L	**2.78**	**0.84**	0.90	4.38	0.86	**0.21**
21	Anchovies	**2.98**	**3.28**	3.00	10.90	0.76	**0.28**
22	Mackerel	**3.68**	**1.16**	0.39	1.98	1.00	**0.20**
23	Squid	**3.64**	**2.10**	0.98	2.70	0.83	**0.36**
24	Butterfish	**3.56**	0.90	1.27	1.98	**0.88**	**0.64**
25	Small pelagics	**3.32**	**0.69**	0.97	4.00	0.89	**0.24**
26	Bluefish S	**3.94**	**0.05**	0.51	18.11	0.94	**0.03**
27	Bluefish M	**4.13**	**0.06**	0.51	3.53	0.67	**0.14**
28	Bluefish L	**4.49**	**0.19**	0.49	1.93	0.14	**0.25**
29	Striped bass S	**3.72**	**0.08**	0.25	23.27	0.78	**0.01**
30	Striped bass M	**3.84**	**0.37**	0.25	6.35	0.19	**0.04**
31	Striped bass L	**3.98**	**0.29**	0.24	3.19	0.20	**0.08**
32	Weakfish S	**3.74**	**0.21**	0.45	13.52	0.93	**0.03**
33	Weakfish M	**3.86**	0.30	0.43	4.22	**0.11**	**0.10**
34	Weakfish L	**3.97**	0.04	0.42	2.45	**0.49**	**0.17**
35	Dogfish S	**4.01**	**0.80**	0.25	1.47	0.79	**0.17**
36	Dogfish L	**4.04**	2.70	0.24	0.61	**0.15**	**0.40**
37	Atlantic cod S	**3.57**	**0.07**	0.48	6.91	0.81	**0.07**
38	Atlantic cod M	**3.87**	**0.15**	0.46	3.49	0.97	**0.13**
39	Atlantic cod L	**4.14**	**0.18**	0.43	2.26	0.96	**0.19**
40	Haddock	**3.64**	0.60	0.45	3.00	**0.61**	**0.15**
41	Hake	**3.71**	**1.25**	1.12	3.85	0.64	**0.29**
42	Croaker	**3.53**	0.82	0.45	0.91	**0.38**	**0.50**
43	Yellowtail flounder S	**3.54**	0.04	1.07	4.41	**0.25**	**0.24**
44	Yellowtail flounder L	**3.46**	0.11	1.10	2.90	**0.47**	**0.38**
45	Summer flounder S	**4.07**	**0.09**	0.56	4.41	0.64	**0.13**
46	Summer flounder L	**4.37**	**0.40**	0.53	2.90	0.48	**0.18**
47	Skate	**3.76**	1.66	0.45	2.40	**0.43**	**0.19**
48	Demersal benthivores	**3.54**	**2.62**	0.45	0.91	0.96	**0.50**
49	Demersal piscivores	**4.05**	**0.85**	0.55	1.21	0.95	**0.45**
50	Demersal omnivores	**3.89**	**2.84**	0.45	0.81	0.87	**0.55**
51	Medium pelagic	**4.45**	0.12	0.45	1.84	**0.07**	**0.24**
52	Coastal sharks	**4.41**	**0.02**	0.20	1.25	0.95	**0.16**
53	Pelagic sharks	**4.49**	**0.05**	0.11	0.69	0.32	**0.16**
54	Large pelagics (HMS)	**4.06**	**0.07**	0.58	6.79	0.83	**0.09**
55	Pinnipeds	**4.36**	**0.06**	0.08	5.50	0.25	**0.01**
56	Baleen whales	**3.43**	0.46	0.04	3.22	**0.04**	**0.01**
57	Odontocetes	**4.34**	**0.46**	0.04	14.30	0.60	**0.003**
58	Seabirds	**4.23**	**0.01**	0.28	9.32	0.42	**0.03**
59	Detritus	**1.00**	52.61			**0.53**	

#### Niche overlap and ecological network analysis

Niche overlap analysis can describe a variety of niche partitioning, in the EwE approach it is focused on the trophic relationships [41]. We generated niche overlap plots focusing on the forage fish species, to evaluate how the input of alosine biomass changes the niche for the group when compared to other species. The niche overlap plots contrast and assign a degree of overlap by pairing species based on the trophic interactions, and are given by prey overlap index, which shows whether the two groups are consuming the same food resource, and predator overlap index, which demonstrates if the two groups are preyed by same predators.

Ecological Network Analysis (ENA) is widely used to compare Ecopath models [49]. We ran ENA to better understand the structure and function of the NEUS LME under contemporary and restored anadromous alosine scenarios. These include trophic level decomposition and keystoneness analysis.

The trophic level decomposition analysis breaks the continuous trophic levels of a functional group into discrete trophic levels *sensu* Lindeman according to Ulanowicz’s approach [35,54]. The analysis shows how many discrete trophic levels each functional group belongs to, and the amount of biomass attributed to each discrete trophic level. It calculates the fractions of the flow from each trophic level through each model group. For example, if an animal has 40% of its diet coming from primary producers, and 60% of it diet coming from first-order carnivores, the corresponding fractions of the flow are attributed to both the herbivore and first consumer levels [41]. We were particularly interested in what trophic level decomposition analysis reveals about how biomass and energy flowed through the trophic network and how biomass transfer differs between trophic levels in each scenario.

The “keystoneness index” refers to a continuous ranking of all functional groups according to the importance of their proximity to a keystone role within the marine ecosystem [40]. All groups present a degree of keystoneness. However, few have a keystone role in the ecosystem. We ran a keystoneness analysis (KS_1_, [40]) comparing the two models to determine whether the changes in biomass indicate differences in the keystone ranking of each functional group, in particular the anadromous alosine.

## Results

**Fig 3:**
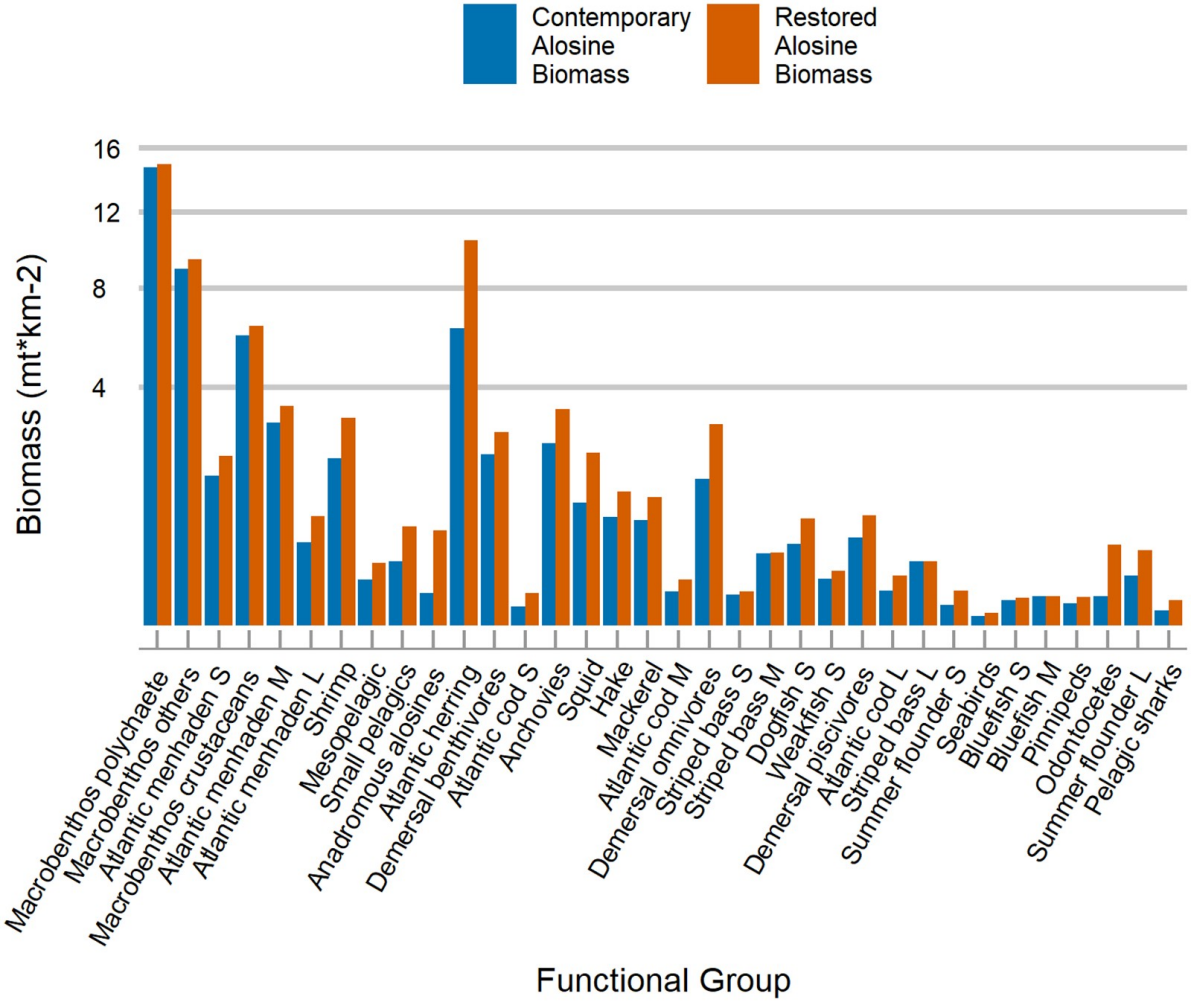
Comparing biomass of functional groups benefiting from alosine restoration. Contemporary and restored biomass for all functional groups impacted by alosine biomass restoration. The y-axis was square transformed to show differences for functional groups with low biomass. Groups that presented biomass change less than to 0.002 mt.km^-2^ were dropped from the graph. Age groups are represented by size, as small (S), medium (M), and large(L).

In the RAB scenario, alosine biomass increased by 137,637 metric tons over the study area, based on production from the three Northern New England watersheds assumed to be fully connected to the sea (Table 3, Fig 3). Thirty-three of the functional groups’ biomasses were left to be estimated by RAB model (Table 2), resulting in 3,603,452 metric tons increase in total biomass over the CAB model, excluding the alosine biomass input. Impacted species were grouped in broader categories as follow: forage species, piscivorous fish, invertebrates and vertebrates (sharks and other species of conservation concern). Besides the anadromous alosine group, the forage species category included mesopelagics (e.g. *Maurolicus* sp.), Atlantic herring, the three size classes of Atlantic menhaden (*Brevoortia tyrannus*), anchovies (e.g. *Ancho* sp.), Atlantic mackerel (*Scomber scombrus*), butterfish (*Peprilus triacanthus*), and other small pelagics (e.g. *Ammodytes* sp.). Butterfish was the only forage species in RAB which the biomass was not calculated by the RAB model (Table 2). For the entire forage species, there was total biomass increase of 1,957,052 metric tons or 50.7%.

**Table 3:** Differences in biomass between the CAB and RAB models.

Node	Group name	CAB Biomass in habitat area (t/km^2^)	CAB Biomass (mt)	RAB Biomass in habitat area (t/km^2^)	RAB Biomass (mt)	Difference between models (mt)	Rate of increase (%)
1	Phytoplankton	20.13	4965306	20.13	4965306	no change	-
2	Bacteria	3.83	943975	3.83	943975	no change	-
3	Microzooplankton	3.16	779699	3.16	779699	no change	-
4	Copepods S	7.81	1926184	7.81	1926184	no change	-
5	Copepods L	7.63	1882771	7.63	1882771	no change	-
6	Gelatinous Zooplankton	1.01	249869	1.01	249869	no change	-
7	Micronekton	7.65	1887951	7.65	1887951	no change	-
8	Macrobenthos polychaete	14.68	3621491	14.92	3680197	58705.556	1.6
9	Macrobenthos crustaceans	5.90	1454319	6.30	1552984	98664.8	6.8
10	Macrobenthos mollusks	8.34	2057161	8.34	2057161	no change	-
11	Macrobenthos others	8.90	2195045	9.39	2316132	121086	5.5
12	Megabenthos filters	3.00	739246	3.00	739246	no change	-
13	Megabenthos others	4.50	1109486	4.50	1109486	no change	-
14	Shrimp	1.96	483458	3.02	744499	261042	54.0
15	Mesopelagic	0.15	37246	0.27	67672	30426	81.7
16	Atlantic herring	6.20	1528349	10.41	2568447	1040098	68.1
17	Anadromous alosines	0.08	18746	0.63	156384	137637	734.2
18	Atlantic menhaden S	1.58	389953	2.02	497511	107557	27.6
19	Atlantic menhaden M	2.88	709874	3.39	835916	126042	17.8
20	Atlantic menhaden L	0.49	120376	0.84	206011	85635	71.1
21	Anchovies	2.32	572244	3.28	808649	236404	41.3
22	Mackerel	0.77	190916	1.16	285025	94108	49.3
23	Squid	1.06	261955	2.10	517432	255477	97.5
24	Butterfish	0.90	221502	0.90	221502	no change	-
25	Small pelagics	0.29	71532	0.69	170676	99144	138.6
26	Bluefish S	0.05	11100	0.05	13444	2344	21.1
27	Bluefish M	0.06	14553	0.06	15160	607	4.2
28	Bluefish L	0.19	47606	0.19	47930	324	0.7
29	Striped bass S	0.07	16325	0.08	19978	3653	22.4
30	Striped bass M	0.37	90113	0.37	91250	1138	1.3
31	Striped bass L	0.29	71047	0.29	72099	1052	1.5
32	Weakfish S	0.16	38233	0.21	52428	14195	37.1
33	Weakfish M	0.30	74739	0.30	74739	no change	-
34	Weakfish L	0.04	8880	0.04	8880	no change	-
35	Dogfish S	0.47	116295	0.80	197361	81066	69.7
36	Dogfish L	2.70	665987	2.70	665987	no change	0.0
37	Atlantic cod S	0.03	6559	0.07	18429	11870	181.0
38	Atlantic cod M	0.08	20620	0.15	36221	15602	75.7
39	Atlantic cod L	0.08	20801	0.18	43216	22416	107.8
40	Haddock	0.60	148737	0.60	148737	no change	-
41	Hake	0.83	203989	1.25	308565	104575	51.3
42	Croaker	0.82	201210	0.82	201210	no change	-
43	Yellowtail flounder S	0.04	10827	0.04	10827	no change	-
44	Yellowtail flounder L	0.11	27417	0.11	27417	no change	-
45	Summer flounder S	0.03	7385	0.09	21288	13904	188.3
46	Summer flounder L	0.18	43273	0.40	97531	54258	125.4
47	Skate	1.66	408226	1.66	408226	no change	-
48	Demersal benthivores	2.05	506644	2.62	646985	140341	27.7
49	Demersal piscivores	0.55	134677	0.85	210712	76035	56.5
50	Demersal omnivores	1.50	369993	2.84	701726	331733	89.7
51	Medium pelagic	0.12	29846	0.12	29846	no change	-
52	Coastal sharks	0.02	4415	0.02	4620	204	4.6
53	Pelagic sharks	0.02	3947	0.05	11233	7287	184.6
54	Large pelagics (HMS)	0.07	17266	0.07	17401	135	0.8
55	Pinnipeds	0.04	8633	0.06	14516	5883	68.1
56	Baleen whales	0.46	114451	0.46	114451	no change	-
57	Odontocetes	0.06	14800	0.46	113977	99177	670.1
58	Seabirds	0.01	1727	0.01	2989	1262	73.1
59	Detritus	52.61	12975694	52.61	12975694	no change	-

**Fig 4:**
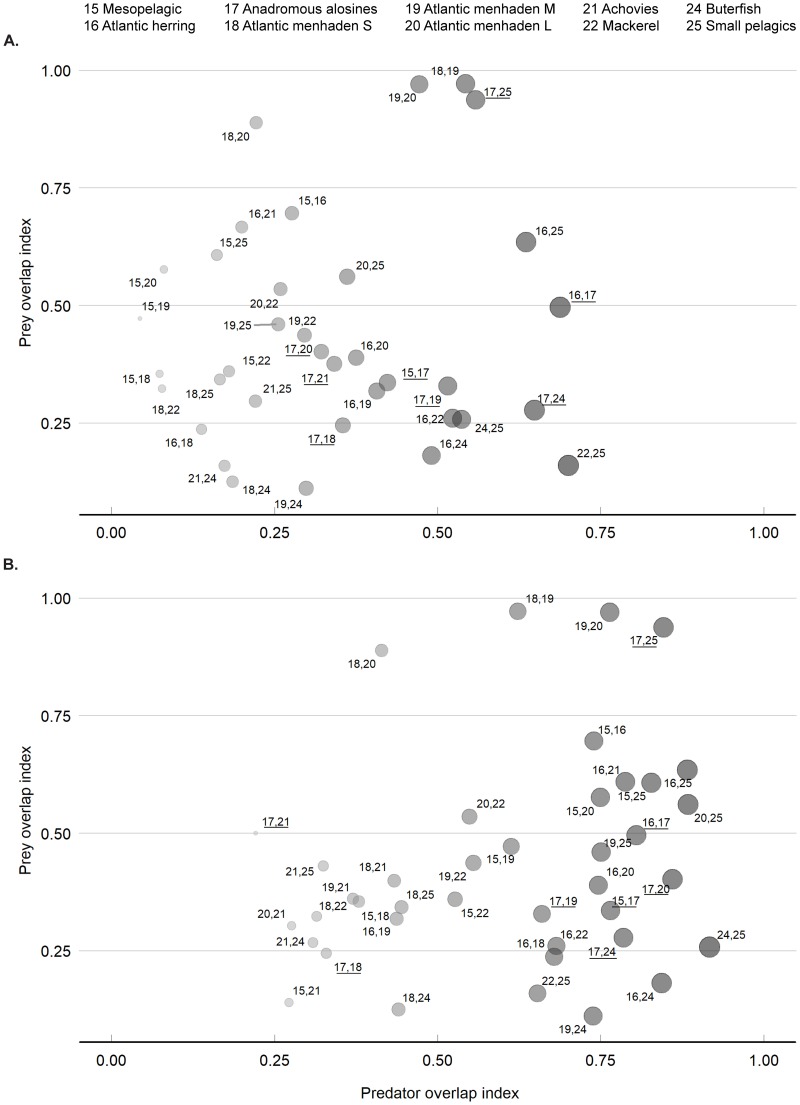
Niche overlap index plot of the forage fish functional groups. (A) Contemporary Alosine Biomass model. (B) Restored Alosine Biomass model. The color gradient and size of nodes are representing the predator overlap index number. Numbers refer to the functional groups, anadromous alosine are represented by underlined numbers.

For both models the forage species groups with the greatest niche overlap where anadromous alosine, other small pelagics, and the three menhaden age classes (Fig 4). There was a considerable shift towards a higher predator overlap index in the RAB model, which was observed among a number of species with the anadromous alosine group (Fig 4). The RAB model indicates stronger predator overlap between anadromous alosines and Atlantic herring, medium and large menhaden, and mesopelagics, demonstrating the potential food base for the main apex predators.

For piscivorous species, including economically important Atlantic cod and summer flounder (*Paralichthys dentatus*), biomass potentially increased by 26.6%, the equivalent of 875,113 metric tons (Table 3, Fig 3). Cod was divided into three size classes, small (≤ 20 cm total length), medium (21–50 cm), and large (>50 cm), to account for ontogenetic stages. Cod biomass increased for all three size groups, but large cod increased the most in the alosine biomass restoration scenario (22,438 mt)––this is roughly equivalent to the entire Gulf of Maine spawning stock biomass from 1980 to 1990 [55]. In addition to changing temperature, another limitation for cod populations is an energetic bottleneck that occurs after age four (large cod > 50 cm), when their shift from a benthic to a pelagic diet caps productivity [56]. Our model suggests that increasing the forage fish base would directly benefit large cod by opening up the bottleneck.

From the invertebrates groups, the RAB model was set to calculate the biomasses for shrimp and squid functional groups, while for macrobenthos and megabenthos we provided the biomass values (S1 File for the list of species). The squid functional group composed by longfin inshore squid (*Doryteuthis pealeii*) and northern shortfin squid (*Illex illecebrosus*), had an increase of 97.5%, the equivalent of 255,477 metric tons. For the shrimp group, there was an increase of 54% or 261,041 metric tons.

**Fig 5:**
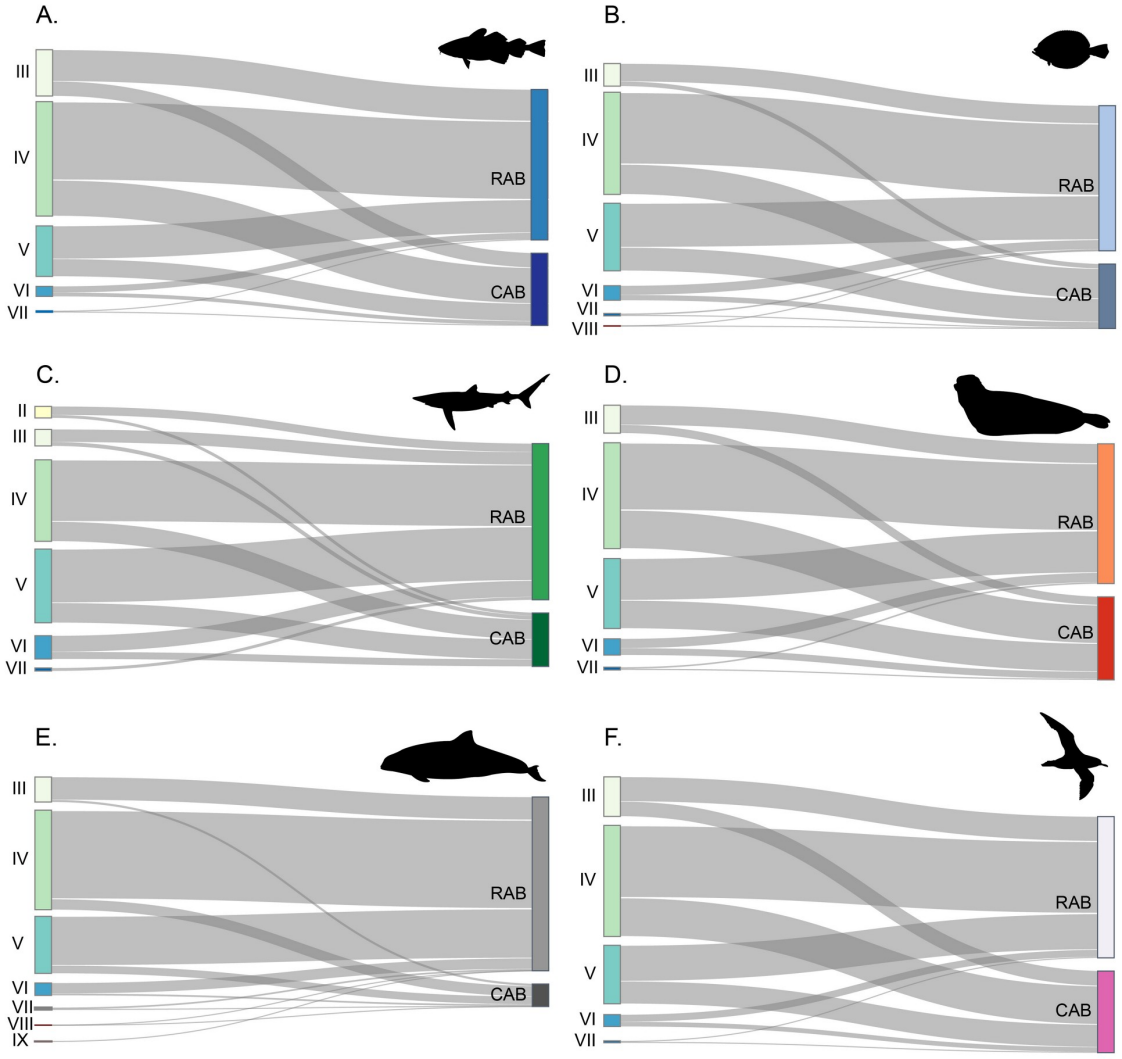
Trophic level decomposition of key species in the Northeast US marine ecosystem. Roman numerals represent the discrete trophic levels of the functional groups in the Contemporary Alosine Biomass (CAB) and Restored Alosine Biomass (RAB) models. (A) Large Atlantic cod, *Gadus morhua*. (B) Large summer flounder, *Paralichthys dentatus*. (C) Pelagic sharks, *Sphyrna* sp., *Carcharodon Carcharias*, *Prionace glauca*, *Isurus* sp., *Lamna nasus*, and *Alopias vulpinus*. (D) Pinnipeds, *Phoca vitulina*, *Halichoerus grypus*, *Pagophilus groenlandicus*, and *Cystophora cristata*. (E) Odontocetes, *Delphinus delphis*, *Globicephala* sp., *Grampus griseus*, *Kogia* sp., *Lagenorhynchus acutus*, *Phocena phocena*, *Physeter macrocephalus*, *Stenella coeruleoalba*, *S*. *frontalis*, *Tursiops truncatus*, *Ziphius* sp. (F) Seabirds, *Calonectris diomedae*, *Fulmarus glacialis*, *Larus marinus*, *L*. *argentatus*, *L*. *philadelphia*, *Oceanites oceanicus*, *Phalaropus fulicarius*, *Puffinus gravis*, *P*. *griseus*, *Rissa tridactyla*, *Sula bassanus*.

Species of conservation concern benefitted from the augmented forage base. Toothed whales, pinnipeds, pelagic sharks and seabirds, together, showed a biomass increase of 69% or 113,948 metric tons. Toothed whales (Odontocetes) alone would potentially increase by 99,177 metric tons. The contrast between the CAB and RAB models trophic level decompositions shows the magnitude of the change in biomass flows between the scenarios. The trophic level decomposition analysis shows the difference in biomass flows from each discrete trophic level and illustrates the differences in the magnitude of the trophic composition of species of conservation concern in NEUS LME, and how the new biomasses increase the allocation of the fractions of the flow. We separated key functional groups to present the magnitude of energy flow changes attributed to increased anadromous alosine biomass, and how the restoration of only a few rivers promotes additional production across multiple key species (Fig 5, Table 4). Table 4 shows the allocations’ differences between CAB and RAB models, used to generate Fig 5.

**Table 4:** The difference in trophic level decomposition (*sensu* Lindeman) between the CAB and RAB models.

			Discrete trophic level (mt.km^-2^.year^-1^)							
Id.	Functional group	Species	II	III	IV	V	VI	VII	VIII	IX
(A)	Atlantic Cod L	*Gadus morhua*		0.045	0.113	0.041	0.007	0.001		
(B)	Summer flounder L	*Paralichthys dentatus*		0.106	0.333	0.164	0.031	0.005	0.001	
(C)	Pelagic sharks	*Alopias vulpinus*		0.002	0.008	0.007	0.002			
		*Carcharodon carcharias*								
		*Isurus* sp.								
		*Lamna nasus*								
		*Prionace glauca*								
		*Sphyrna* sp.								
(D)	Pinnipeds	*Cystophora cristata*	0.001	0.026	0.067	0.032	0.006	0.001		
		*Halichoerus grypus*								
		*Pagophilus groenlandicus*								
		*Phoca vitulina*								
(E)	Odontocetes	*Delphinus delphis*		0.790	2.991	1.576	0.337	0.053	0.007	0.001
		*Globicephala* spp.								
		*Grampus griseus*								
		*Kogia* spp.								
		*Lagenorhynchus acutus*								
		*Phocena phocena*								
		*Physeter macrocephalus*								
		*Stenella coeruleoalba*								
		*S*. *frontalis*								
		*Tursiops truncatus*								
		*Ziphius* spp.								
(F)	Seabirds	*Calonectris diomedae*		0.008	0.027	0.011	0.002	0.001		
		*Fulmarus glacialis*								
		*Larus marinus*								
		*L*. *argentatus*								
		*L*. *philadelphia*								
		*Oceanites oceanicus*								
		*Phalaropus fulicarius*								
		*Puffinus gravis*								
		*P*. *griseus*								
		*Rissa tridactyla*								
		*Sula bassanus*								

**Fig 6:**
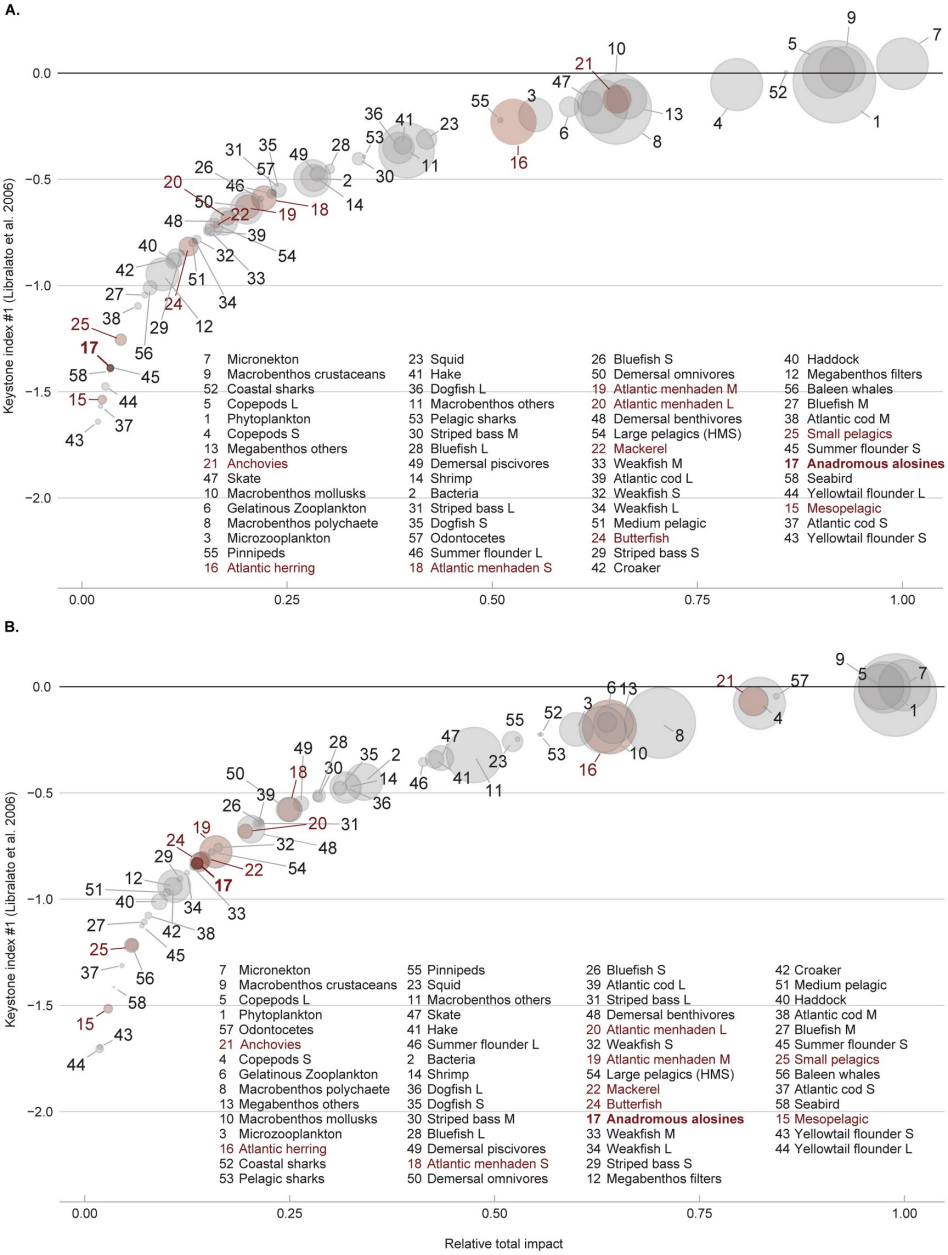
Keystoneness analysis for both models using KS_1_ index. The functional group lists are ranked and ordered in terms of keystoneness, and circle size reflects biomass. (A) Keystoneness and biomass for the CAB, (B) keystoneness and biomass for the RAB model. Forage fish species are highlighted in red, and anadromous alosine group is in bold.

The keystoneness analysis, a measure of network connectivity, also revealed differences between the two models. For the CAB model, the top five species ranked from highest to lowest on the keystone index were: micronekton (0.044), macrobenthos crustaceans (0.017), coastal sharks (0.0039,), large copepods (0.0032) and phytoplankton (-0.041). The RAB model’s first- and second-ranked functional groups were the same as the CAB model (micronekton = 0.00668, and macrobenthos crustaceans = -0.00124); however, large copepods (-0.00389) and phytoplankton (-0.0393) occupied the third and fourth places, respectively, and Odontocetes (-0.0463) occupied fifth place (Fig 5). Among the groups under the forage fish category, the anadromous alosine group was the one that showed the most considerable changes in keystoneness index, increasing twelve positions on the rank, from fifty-third place on CAB to forty-first place on RAB model. Anchovies were the component of the forage fish species that ranked the highest, with a rise of two steps on the keystoneness ranking (CAB KS_1_ = -0.123 [rank 8], and RAB KS_1_ = -0.067 [rank 6]). Atlantic herring also showed a rank increase of two steps, shifting from the fifteenth position to thirteenth (CAB KS_1_ = -0.23, and RAB KS_1_ = -0.201) (Fig 6).

## Discussion

The Restored Alosine Biomass model offers a “what if” scenario of potential benefits to NEUS LME due to increased connectivity between rivers and oceans. Since anadromous alosine group depletion is acknowledged and its restoration is an active management goal, modeling the potential ecological benefits of much larger alewife populations will inform ongoing efforts. Our approach incorporated EE parameters from the CAB model to generate biomass potential for functional groups that have trophic interactions with the anadromous alosine group. Our results, based solely on alewife biomass changes, highlights the species importance as a component of the forage fish complex. This effort represents the first-time historical landscape-based estimates of an anadromous fish species were used to inform a marine ecosystem model. Increasing overall forage group biomass promoted energy flow through the mid-trophic levels to the benefit of numerous functional groups, demonstrating the enhanced potential of ecosystems with river-ocean connectivity. Ongoing efforts to advance understanding of ecosystem connectivity should be encouraged, due to the widespread positive impacts in the current simulation.

Keystone species are essential drivers of ecosystem processes and can impose limits on other species through predation or resource partitioning. Predators have more substantial ecosystem impacts relative to their biomass and drive top-down control of the system [57]. Comparing the RAB and CAB models the top keystone species remained similar, with the downgrading of coastal sharks and upgrading of odontocetes (dolphins, porpoises and sperm whales). In Newfoundland, the Mediterranean and the Eastern Pacific odontocetes also rank high on the keystoneness index [40,58,59]. Coll *et al*. [58] attribute the group’s significance to its non-exploited status. Anadromous alosines group had the second highest ranking increase, highlighting how the group’s ecosystem roles were shuffled as their abundance waxes. In both scenarios, phytoplankton and zooplankton components, such as micronekton and copepods, ranked high in keystoneness (Fig 6). Other models [1,2] have demonstrated the pervasive influence of seasonal phytoplankton regimes in temperate and coastal ecosystems such as the Gulf of Maine and the Chesapeake Bay.

Regardless, the approach allows assessment of how alosines are connected to broader ecosystem functioning through trophic relationships, and offers a perspective on how increases in the contribution of forage fish will impact top predators and energy flows [60]. Previous studies point out that different dynamics are possible in ecosystems, such as top-down, bottom-up control, and wasp waist fishery dynamics [61]. Although none of the groups of the forage fish complex are considered marine keystone species, their role in energy transfer is relevant to the functioning of the NEUS LME. An order of magnitude change in alosine biomass positively drove potential flow to species of economic and conservation concern. In an ecosystem, several variables can also affect biomass fluctuations including climate fluctuations, fishing pressure, geographic dispersal of species and changes in productivity pulses that were not accounted for in this simulation and would have to be considered when operationalizing such models for ecosystem-based fisheries management. To do so will require a perspective that includes both the connectivity to freshwater ecosystems and the historical productivity estimates, if the full potential of fisheries is expected.

**Fig 7:**
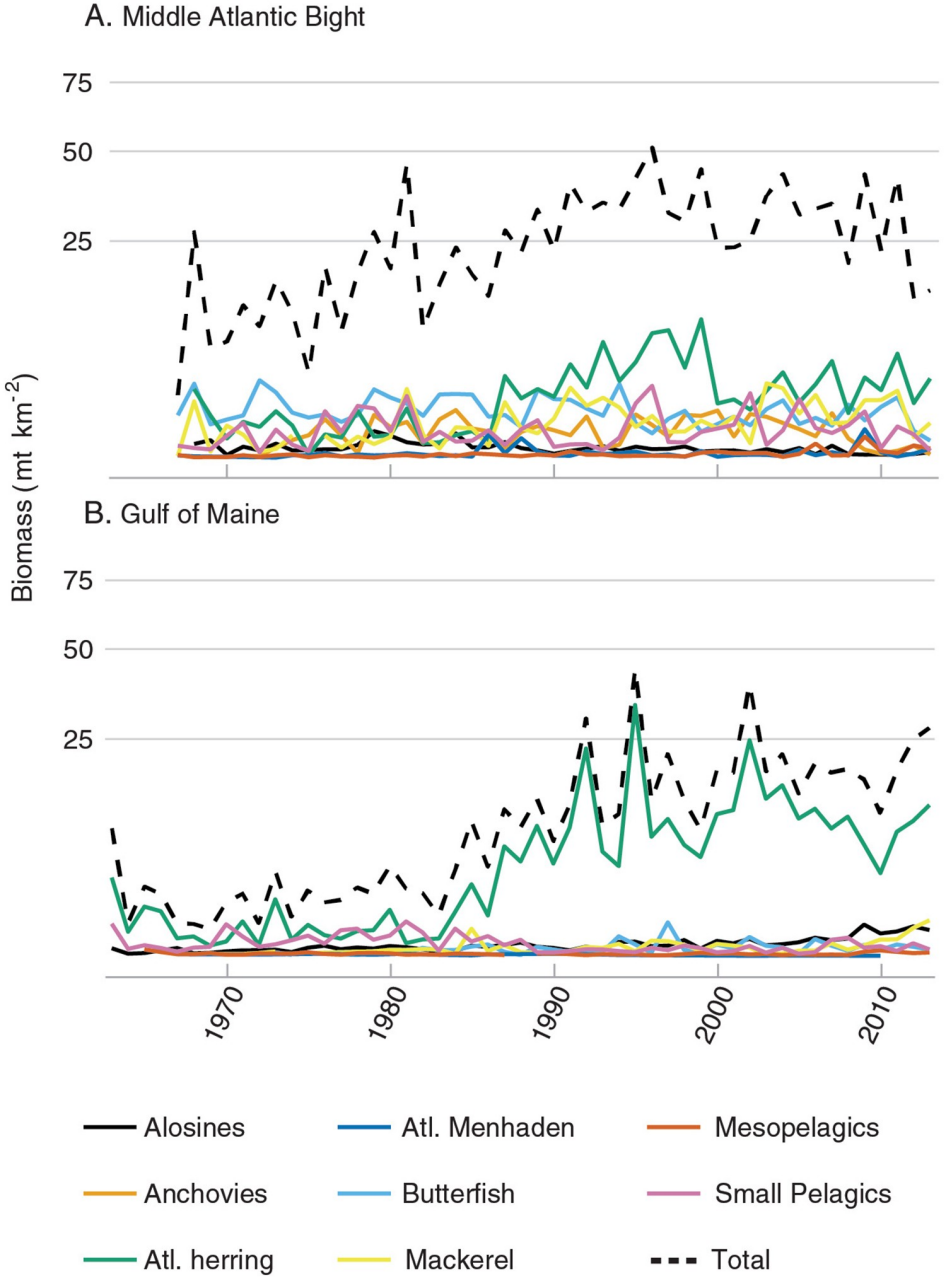
Contrasting forage fish biomass time series in two Northeast US sub-regions. (A) In the Middle Atlantic Bight (MAB), the total forage fish biomass trend is driven by similar fluctuations within several different forage fish stocks. (B) In the Gulf of Maine (GOM), the total forage fish biomass trend is mostly driven by Atlantic herring (green line) fluctuations. Biomass data is from NEFSC trawl surveys, 1963 to 2013, with corrected catchability (q).

Current river herring stocks are but remnants of historically abundant and widespread populations [25]. Their absence from coastal ecosystems contributes to a niche-specific bottleneck in pelagic mid-trophic forage species group. As climate change places more energetic demands on predator populations, loss of functional redundancies in prey populations will become even more problematic as the remaining forage species undergo natural fluctuations [7,16]. In diverse ecological communities, seasonal pulses of prey species with different life histories provide stable food for apex predators (Fig 7A). This portfolio [62] effect no longer appears to function in the Gulf of Maine, which has become heavily reliant on Atlantic herring, and predators likely suffer higher energetic costs during periods of low Atlantic herring abundance (Fig 7B). In addition to the impacts on the marine environment, the loss of connectivity also affects riverine [23] and estuarine systems. There is evidence that juvenile planktivorous, such as Atlantic herring and sand lance are more dominant food base than river herring in the estuary of Saco River [63], a heavily dammed watershed, adjacent to the watersheds of our study. Atlantic herring stock projections show a high likelihood of overfished and overfishing status in the future, due to sustained low recruitment since 2011 [64]. This raises concern for the sustainability of the forage base and their fisheries in the Gulf of Maine.

Stabilizing the forage fish portfolio requires re-establishing species diversity across the ecosystem. We acknowledge the likelihood that fish stocks will continue to be managed individually, yet our work emphasizes that even depleted stocks are critical to the forage fish pool [65]. Restoring diversity requires restoring connectivity across the entire spatiotemporal patchwork. Managing the pelagic forage complex as a group is analogous to the current groundfish framework, which considers co-occurring species with separate assessments but with a recognition of similarities in habitat-use, fisheries catch and functional roles in the ecosystem.

Large fluctuations in fish populations have led to the assumption that populations always self-replenish along taxa-specific time scales [66]. Marine clupeids are more likely to experience population recovery on shorter timescales than gadids and other marine fishes [66], and one would think that small pelagic anadromous fish are the same. However, lack of population recovery for clupeids stocks such as American shad and river herring suggests that resilience of the anadromous forage fish complex has been overestimated concerning the multiple impacts they face [67].

Despite recognizing the importance of the forage group as a vector of energy to higher trophic levels, there is a lack of understanding of the spatial-temporal dynamics of different forage species. Currently, small pelagics account for 30% of global fisheries landings. Atlantic herring and menhaden yield the highest landings among all fish species in the Northeast United States [68]. They support several fisheries sectors, including the bait, feed, and oil reduction and extraction industries. However, rates of forage fish exploitation are raising red flags as their depletion is linked to the poor body condition, decreased fecundity, impeded recovery, and threatened the survival of a wide range of species [1,69]. Coastal and anadromous species are important constituents of the forage fish group, as we have demonstrated, yet they have experienced even higher rates of decline [70]. The functional removal of Atlantic herring in the 1970s [71], following declines in river herring and Atlantic menhaden, would have considerably strained remaining forage populations, such as sand lance [72].

Hilborn *et al*. [15] point out that predators often have flexibility in foraging; only 10% of predator populations are directly linked to a single prey species. We find that the MAB region is more likely to promote generalist diets than the GOM. As a result, natural fluctuations in forage fish abundance [65,73] in MAB are more easily offset by redundancy in the forage base than in the GOM, where predators have become dependent on Atlantic herring (Fig 7). McClatchie *et al*. [73] show that, despite naturally fluctuating cycles of the three main forage species pre-exploitation, their aggregate biomass held constant. Unfortunately, most diet information aggregated [74] and collected over a limited seasonal period. Thus, seasonal dependence on specific forage species is often underestimated. However, there are plenty of examples of species that heavily rely on short bursts of single prey species [9,17,75]. A new paradigm is emerging, which considers spatial and temporal variations in the forage base, and contrasts availability versus food quality in predator diets. Simplified food web models and diet aggregations can underestimate the importance of forage fish in food webs [76], and scarce information may limit the successful application of management policies intended to provide a more holistic approach. The value of alosine clupeids is made even greater by their niche overlap, making them a flexible food item for many species at specific times and places.

Restored watersheds with incentivized dam removal and fish passage policies will raise the capacity of resilience of anadromous forage fish populations. Applying these measures, we can once again provide the benefits of the successful anadromous life history strategy that became disadvantaged with anthropogenic modifications to the environment [67]. We acknowledge that dam removal should be examined as a case by case, weighing the trade-offs that might occur from removing the services associated with the dams [77]. Here we quantified the potential of river restoration and tested the potential biomass flow increase in marine food webs. We highlight the historical role of rivers in marine ecosystem functioning through anadromous forage fish, a group that requires a myriad of habitats to support their life history strategies [67].

We acknowledge that there is no way back to Neverland, or to past conditions, as changes in the physical system guide biological process away from the reference points [78]. However, we should consider historical baselines to avoid the use of already impacted populations and ecosystems reference points and parameters to identify targets for rehabilitation measures, the essence of shifting baseline syndrome [79], and establish a clear path towards management goals. In the end, our motivation to perform the current study came from centuries of historical accounts of the importance of alewife schools in attracting highly priced “good fish” [80]. Ongoing efforts to advance understanding of ecosystem connectivity should be encouraged. Moving forward, a continued conversation regarding all the factors that influence alosine recovery, and other coastal forage populations, and what the ecosystem implications are within a temporal and spatial framework is required for a more holistic approach to managing these coupled natural-human systems.

## Supporting information

S1 File Model documentation. Table and figures for all taxa components of the functional groups and their respective data sources of the NEUS LME.
(DOCX)

S1 Table Contemporary Alosine Biomass (CAB) model diet matrix. (XLSX)

S2 Table Restored Alosine Biomass (RAB) model diet matrix. (XLSX)
