# Supplementary material for: Opening the tap: Increased riverine connectivity strengthens marine food web pathways
Source: PLoS One. 2019 May 23;14(5):e0217008. doi: 10.1371/journal.pone.0217008 (PMC6532889; doi:10.1371/journal.pone.0217008)
Supplement: S1 File — Table and figures for all taxa components of the functional groups and their respective data sources of the NEUS LME. (DOCX) [file pone.0217008.s001.docx]

**Supporting Information**

Title: Opening the tap: Increased riverine connectivity strengthens marine food web pathways.

Short Title: Connectivity strengthens marine food webs.

Beatriz S. Dias, Michael G. Frisk & Adrian Jordaan

The data used to build the Ecopath with Ecosim model came from the Northeast Fisheries Science Center (NEFSC NOAA) bottom trawl survey, Atlantic States Marine Fisheries Commission (ASMFC) stock assessments, Virginia Institute of Marine Sciences (VIMS), Ecosystem Modelling Analysis eXercise (EMAX), and peer-reviewed publications.

The EMAX project [1,2] generated a comprehensive database for the four sub-regions of the Northeast US large marine ecosystem (NEUS LME): the Gulf of Maine, Georges Bank Southern New England, and Middle Atlantic Bight. Scientists, from diverse disciplines such as physical oceanography, biology, and the social sciences, generated parameters for 36 functional groups within the region. Our base model (CAB) expanded the EMAX project’s functional group resolution, *sensu* Link *et al.* (2006, 2008). We also employed the same time scale (year block 2000 covered the years1996 to 2000), however we used a weighted average of the input parameters (B, P/B, Q/B, and diets) to build our models for the NEUS LME (246,662 km^2^), incorporating stock assessment information for additional groups.

Catch data were obtained at <http://www.st.nmfs.noaa.gov/commercial-fisheries/index> and <http://www.st.nmfs.noaa.gov/recreational-fisheries/index>. We summed species-specific landings from all the coastal states from Maine to North Carolina, and aggregated gear types into seven distinct categories: dredge, trawl, traps, gillnets, purse seine, recreational, and other (gear that did not fit elsewhere). We used stock assessment data for managed species due to its superior resolution. Eight functional groups were divided into size classes to account for ontogenetic differences, using catch-at-age data from stock assessments to determine landings and discards.

Diets for nodes 2-15 and 52-59 were obtained from the EMAX model [1]. For fished species, we acquired diet data from NEFSC trawl surveys and through the VIMS fish food habits database, which includes diet data from the Northeast Area Monitoring and Assessment Program (NEMAP) and the Chesapeake Bay Multispecies Monitoring and Assessment Program (ChesMMAP). For functional groups divided into size classes, we used a size selectivity approach developed by Andre Buchheister and colleagues [3,4] in parallel collaboration with our work.

We used PREBAL analysis prior to balancing (Fig A) to find violations in our Ecopath assumptions (Link (2010) describes this approach).


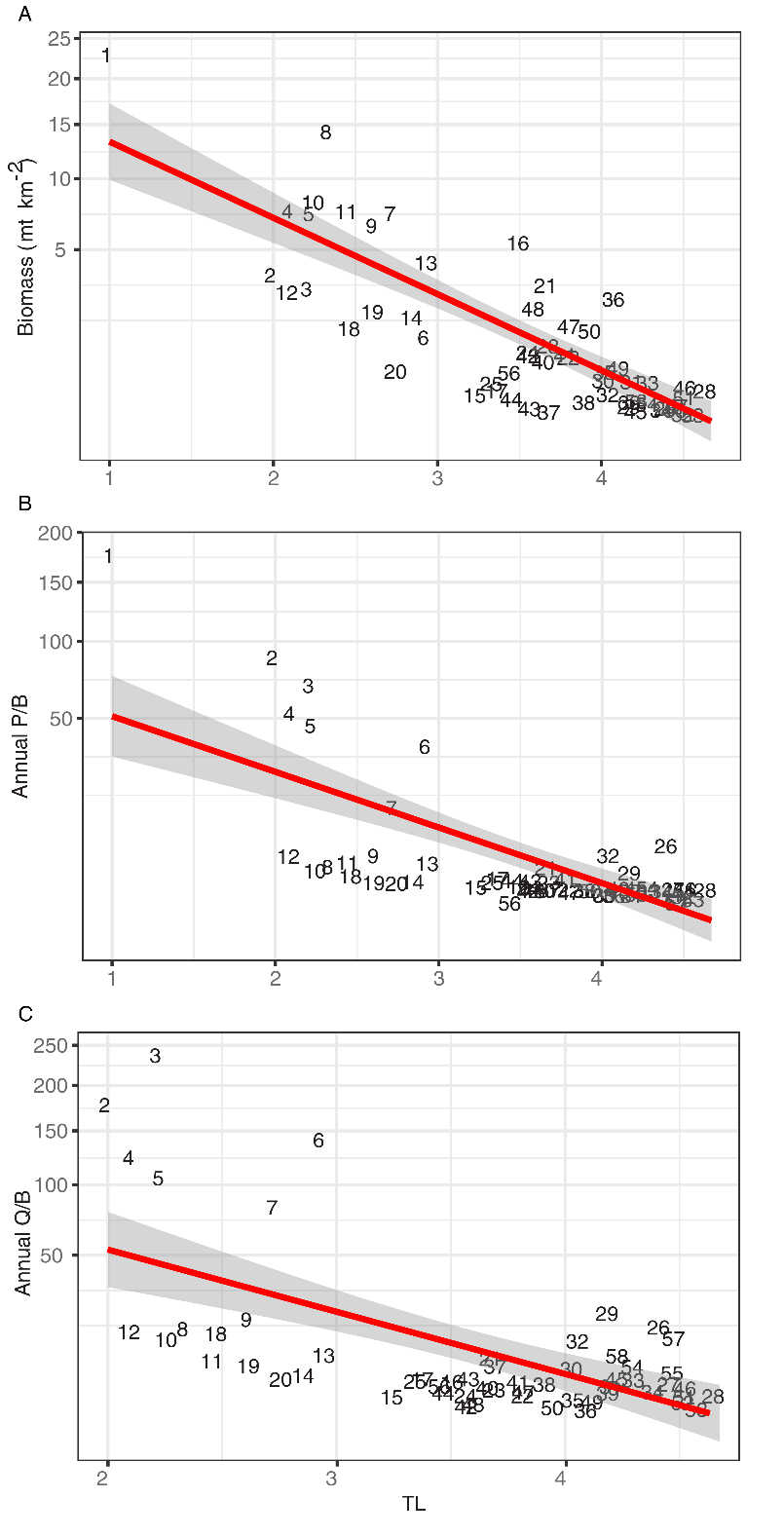


**Fig A. Pre-balancing visual output.** (A) Trophic decomposition (trend line) for biomass. (B) Annual Production per biomass. (C) Consumption per biomass. Proximity to the trend line shows that starting input values are as expected. The numbers represent the nodes shown in the model documentation bellow.

For each estimate, we classified source and data quality using the EwE pedigree routine. To assess uncertainty around parameters of both the Contemporary (CAB) and the Restored Alosine Biomass (RAB) models, we resorted to ensemble parametrization. This technique generates a series of ecopath models using data pedigree to determine the confidence interval scoring for each point parameter [6,7]. Kearney’s createensemble method [8], part of the ecopath_matlab implementation, generated 10000 interactions for Biomass, PB and Q/B ratios, using the lognormal distribution with mean (*x*) and variance (*[pedigree.x/2]^2^*) (Figs B to G). It is important to note that the ensemble analysis was executed only for the input parameters, not the parameters generated by the ecopath model, as we don’t have the pedigrees associated with the estimates (e.g. Figs B and E difference in the number of functional groups). The P/B and Q/B ratios remained the same for both models, and the plots are a replication.


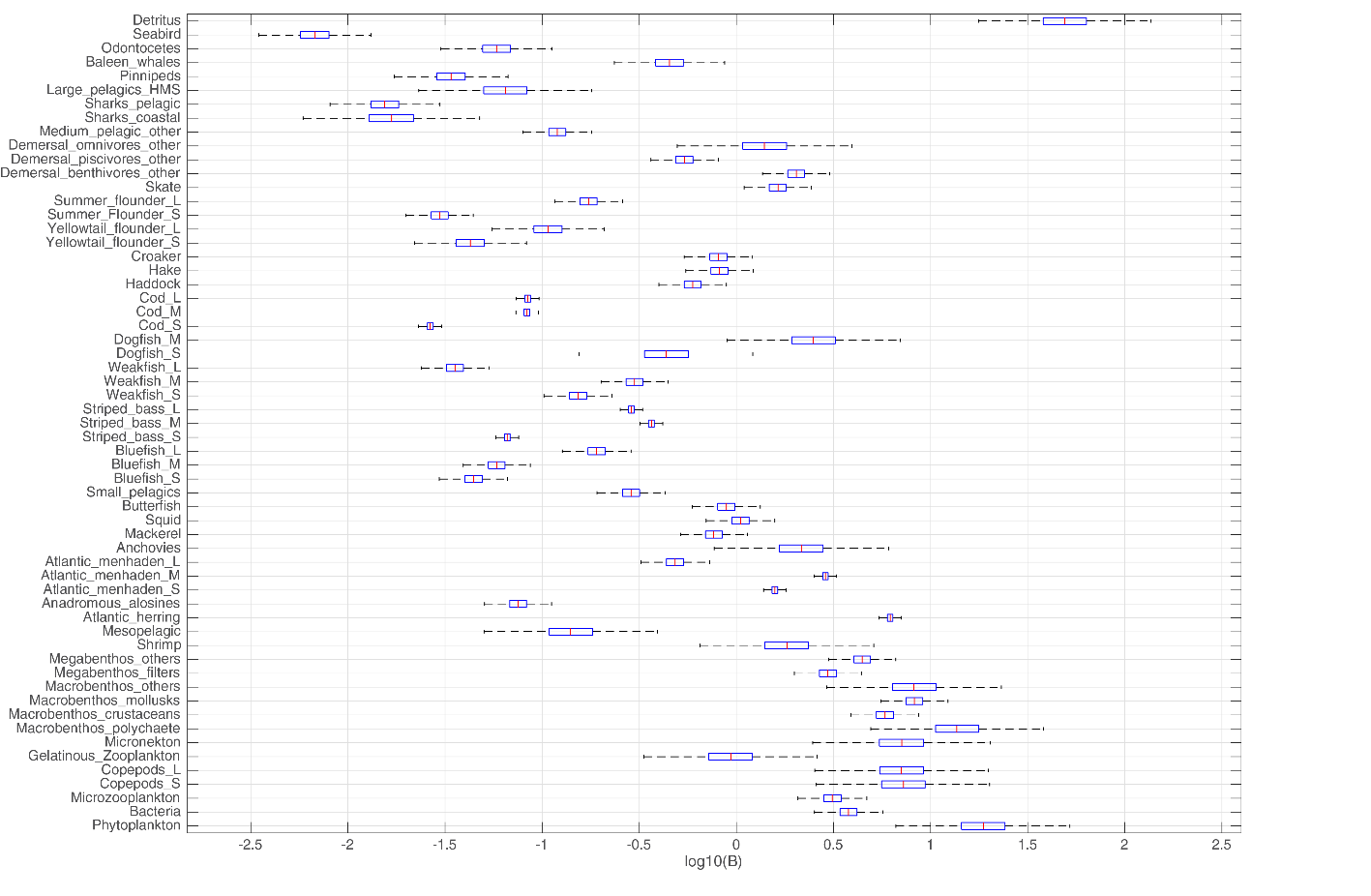


**Fig B. Contemporary Alosine Biomass (CAB) model biomass ensemble plot.** For better visualization, the x-axis scale is logarithmic (base 10).


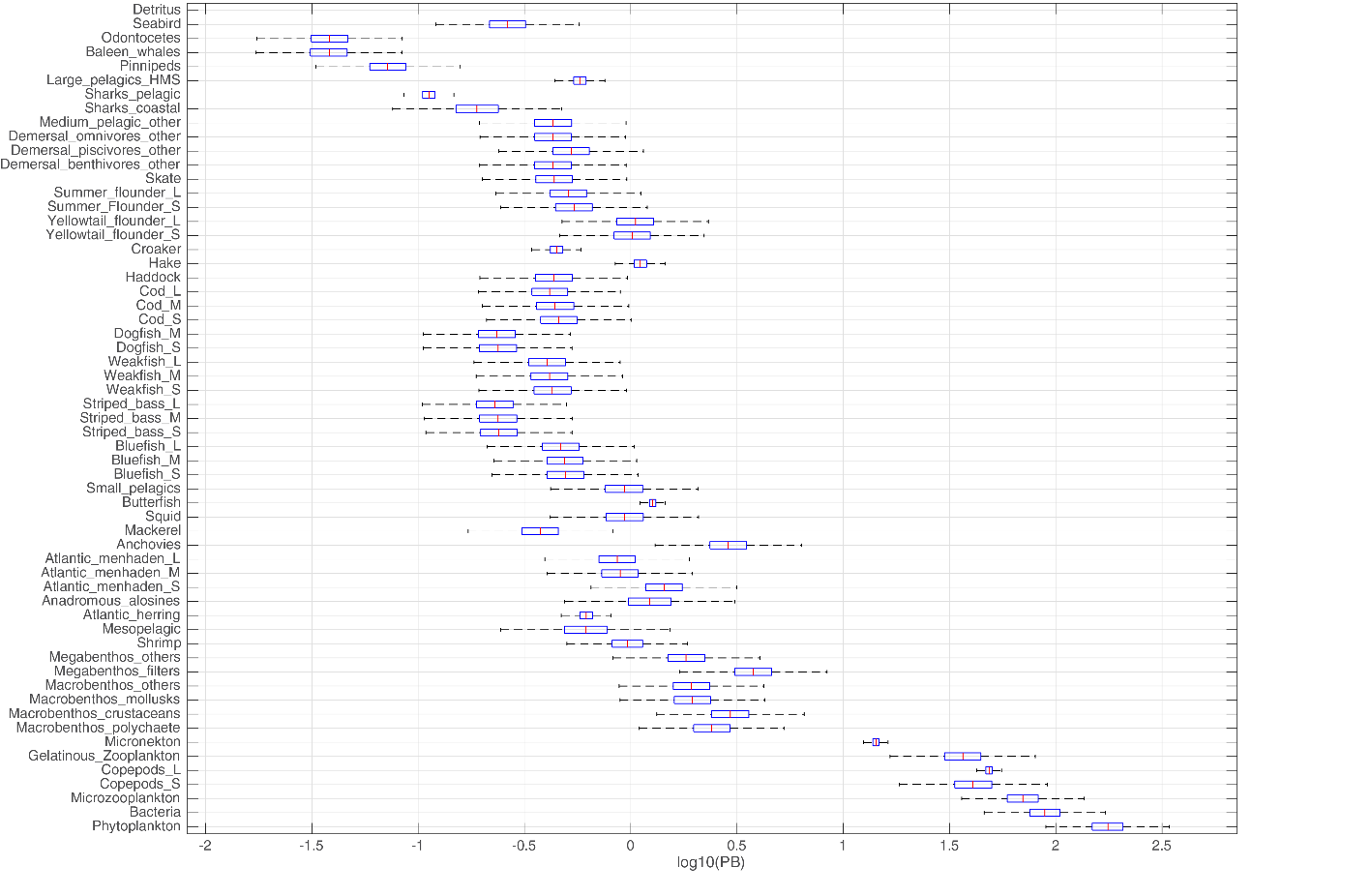


**Fig C. Contemporary Alosine Biomass (CAB) model P/B ratio ensemble plot.** For better visualization, the x-axis scale is logarithmic (base 10).


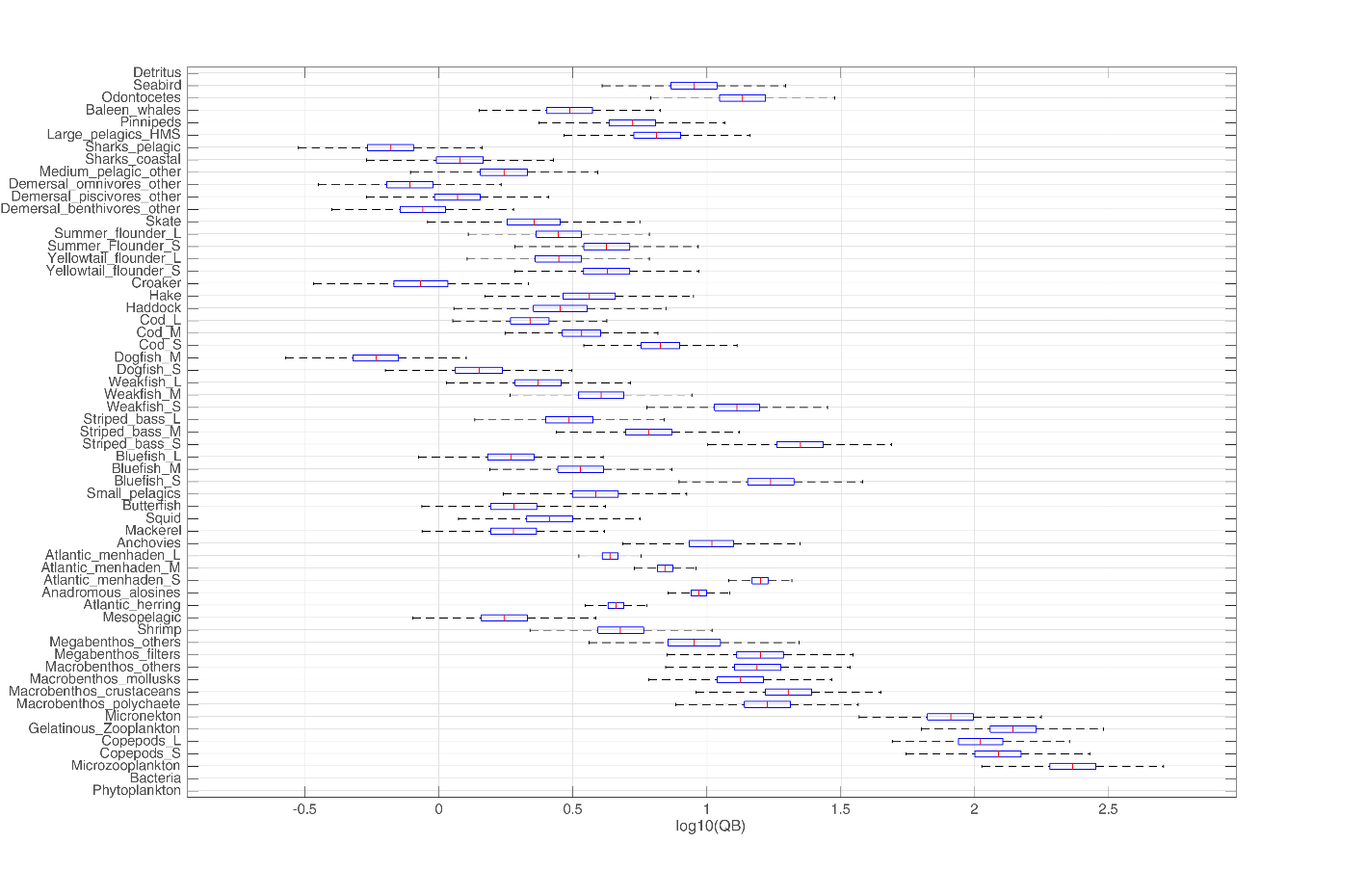


**Fig D. Contemporary Alosine Biomass (CAB) model Q/B ratio ensemble plot.** For better visualization, the x-axis scale is logarithmic (base 10).


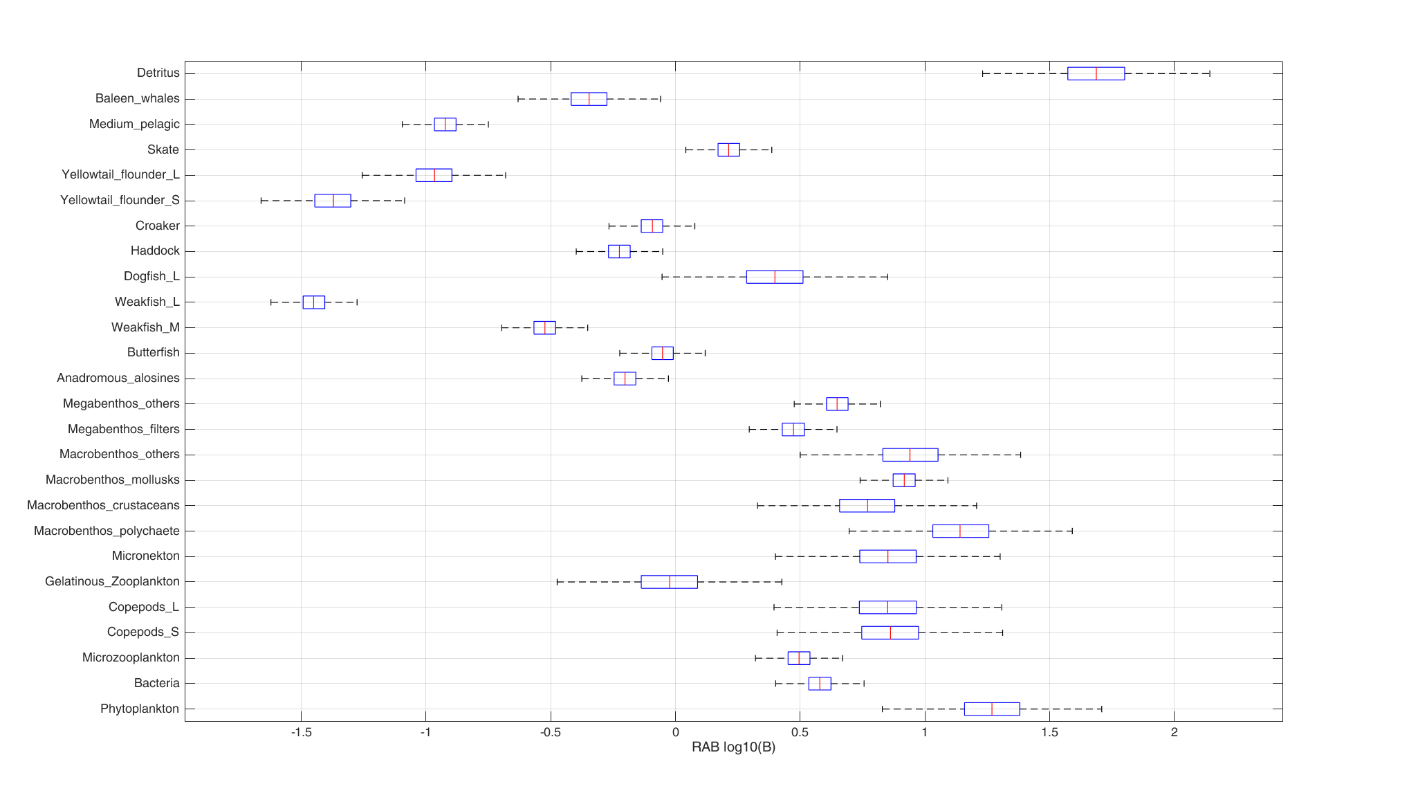


**Fig E. Restored Alosine Biomass (RAB) model biomass ensemble plot.** For better visualization, the x-axis scale is logarithmic (base 10). Groups shown depict all biomass input parameters.


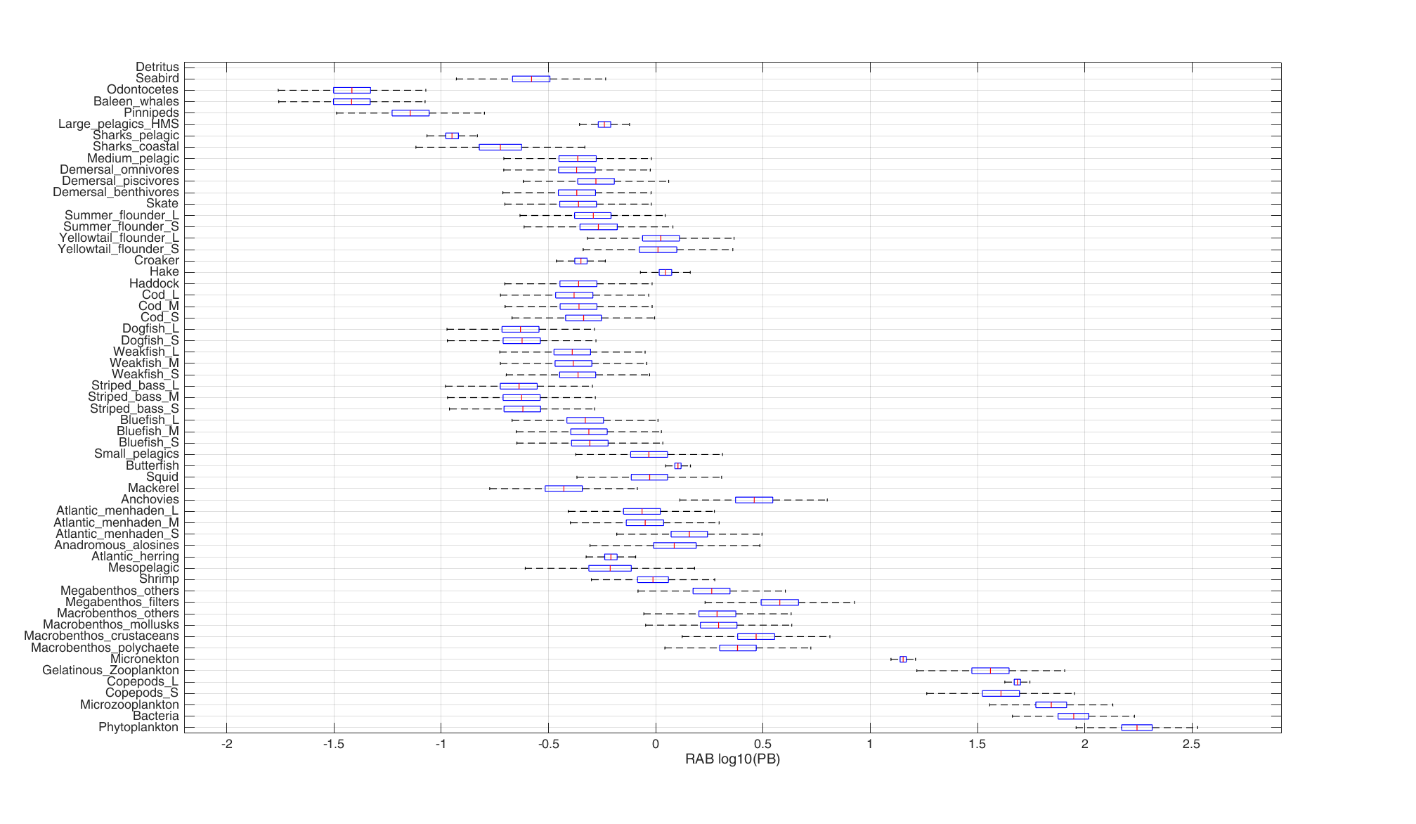


**Fig F. Restored Alosine Biomass (RAB) model P/B ratio ensemble plot.** For better visualization, the x-axis scale is logarithmic (base 10). Results equal the CAB model results.


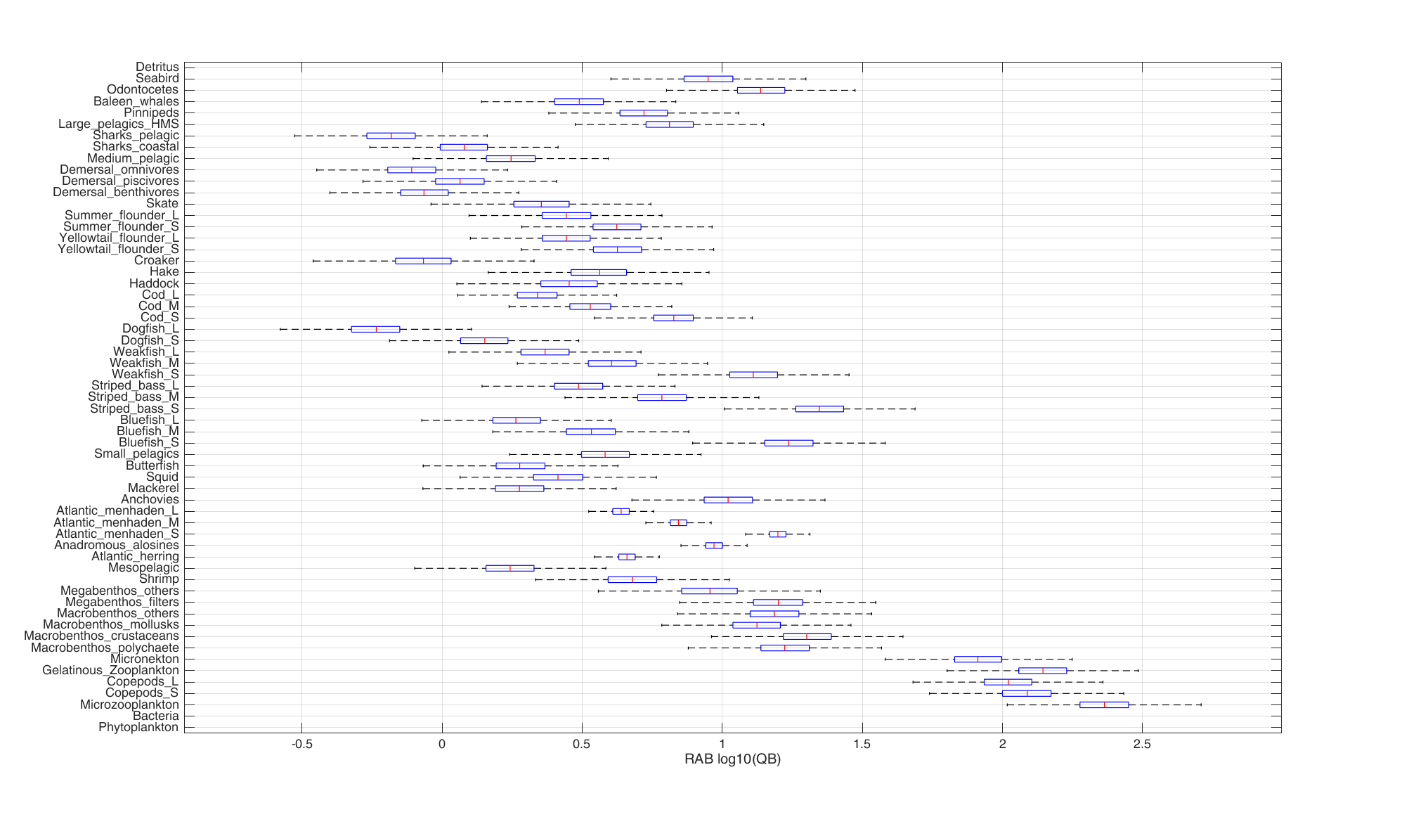


**Fig G. Restored Alosine Biomass (RAB) model Q/B ratio ensemble plot.** For better visualization, the x-axis scale is logarithmic (base 10). Results equal the CAB model results.

Mixed trophic impact analysis shows the effects that biomass changes of determined group has upon the other groups in the system[9]. Diets were not substantially changed, therefore the CAB and RAB mixed trophic impacts (MTI) matrices are not substantially different (Figs H and I).


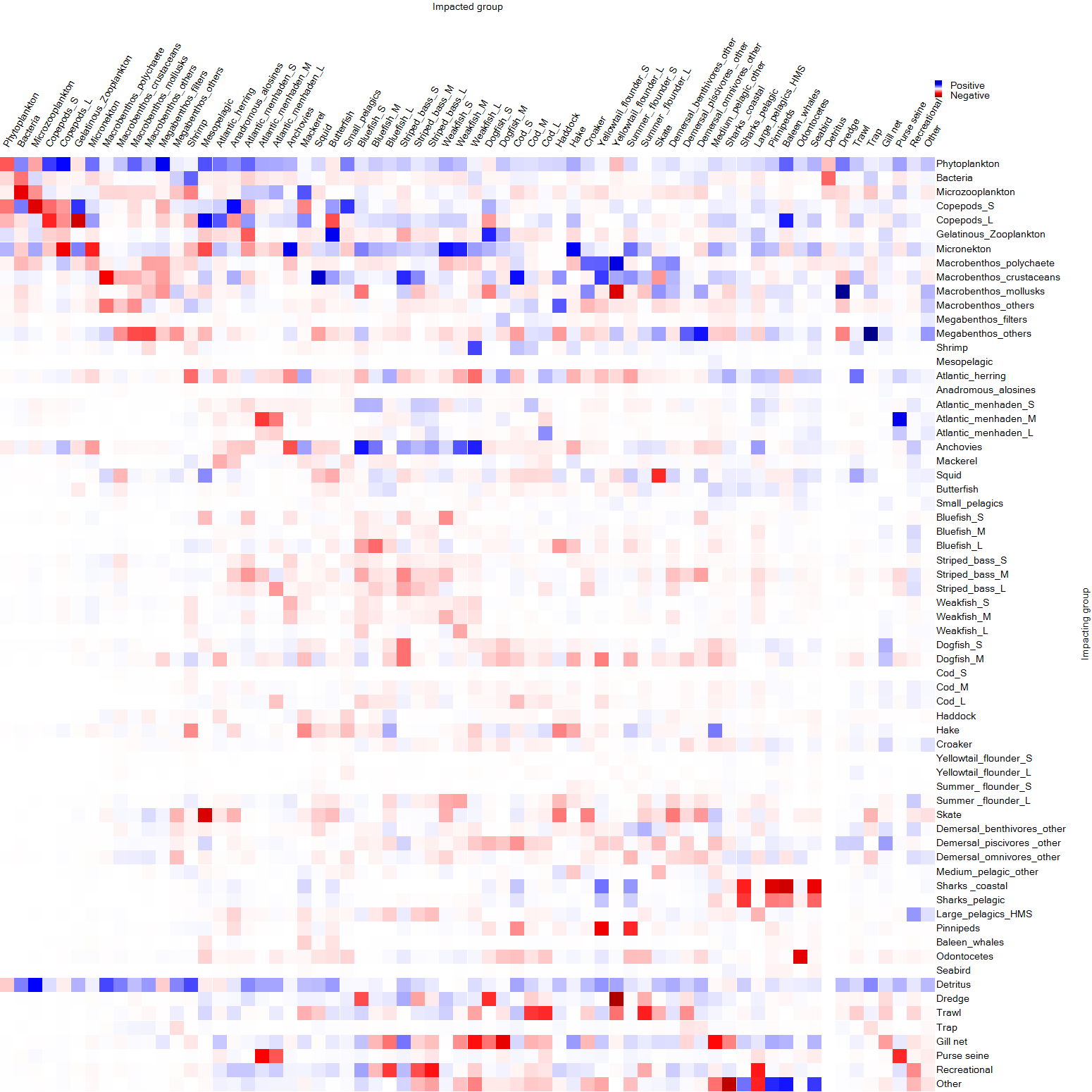


**Fig H. Contemporary Alosine Biomass (CAB) Mixed Trophic Impacts.**


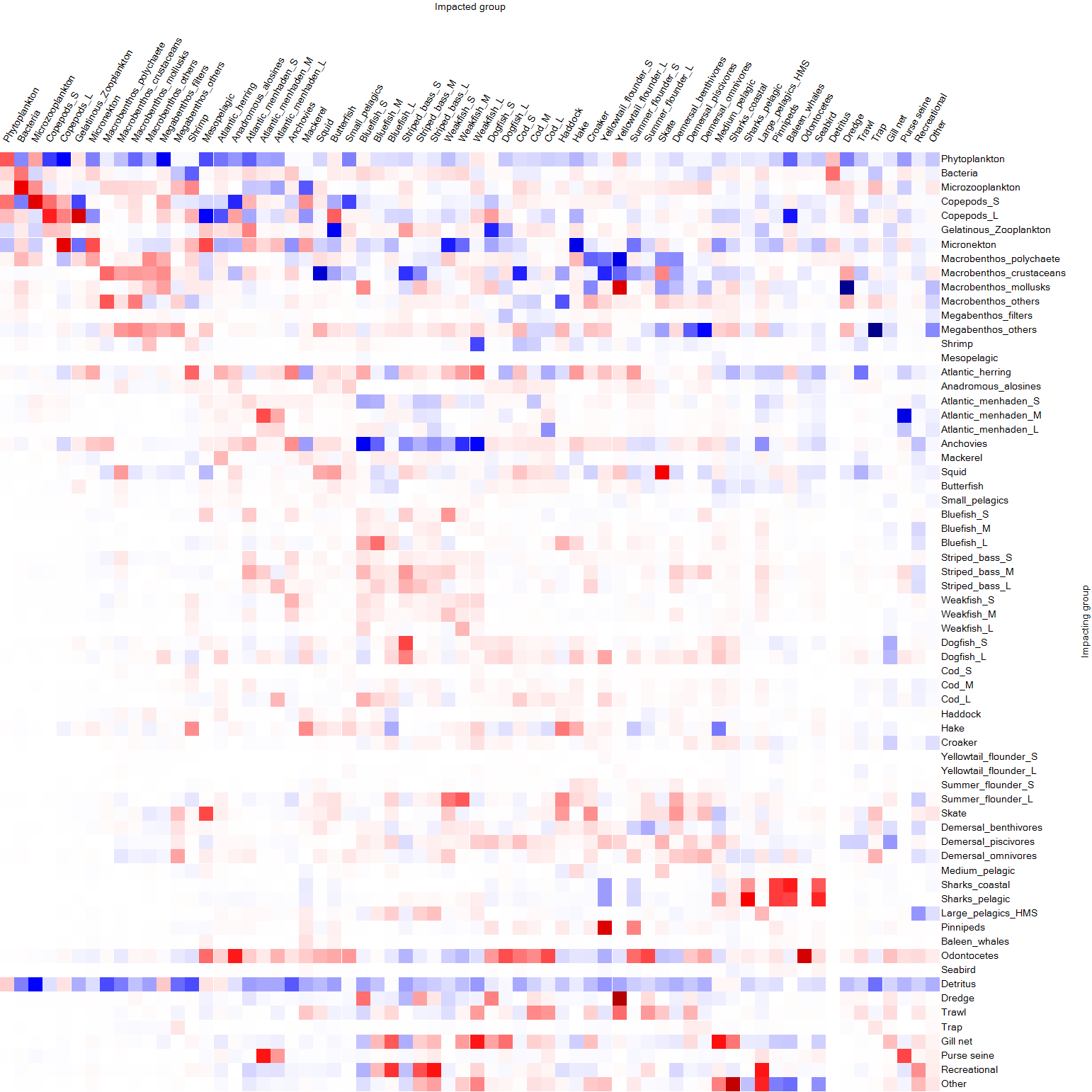


**Fig I. Restored Alosine Biomass (RAB) Mixed Trophic Impacts.**

**Model documentation**

The model documentation applies for the Contemporary Alosine Biomass (B, P/B, Q/B, and Diet) and Restored Alosine Biomass (P/B and Q/B) model parameters. Ecotrophic efficiencies (EE) for RAB model were obtained from CAB model output.

**Node 1. Phytoplankton**

Phytoplankton functional group parameters were obtained from EMAX model initial input data by combining the total biomass of all regions and dividing by the total area (246662 km^2^). Biomass was estimated to be 20.13 mt.km^-2^, while P/B (yr^-1^) input was 180.7. Estimates were derived from *in situ* vertical profiles of chlorophyll a and SeaWiFS remote sensing data. Node calculations are found in Link *et al.*[1].

**Node 2. Bacteria**

Bacteria biomass (3.827 mt.km^-2^) and P/B (91.25) were derived from EMAX models. P/Q (0.5 yr^-1^) was obtained from the EwE user guide [9] and Heymans *et al.* [10]. We generated diet data from the EMAX models using weighted averages from each sub-region (combining the total diet of all regions and dividing by the total area, 246662 km^2^).

**Node 3. Microzooplankton**

The microzooplankton functional group includes protozoa, ciliates, flagellates, and larval stages of benthic invertebrates (meroplankton) [1,3]. Biomass (3.16 mt.km^-2^) and P/B (71 yr^-1^) were estimated from EMAX initial inputs, and Q/B (242.42 yr^-1^) was derived from the EMAX balanced estimate. Diet data were estimated using the methods described for Nodes 1 and 2.

**Nodes 4 and 5. Copepods S and L**

Small copepods comprise the stage I-IV copepodites of large copepod species, and the I-VI copepodites stages of *Centropages hamatus*, *Pseudocalanus* spp., *Temora longicornis*, *Paracalanus parvus*, *Nannocalanus minor*, and *Clausocalanus arcuicornis*. Large copepod species include V and VI copepodites stages of *Calanus finmarchicus*, *Metridia lucens*, and *Centropages typicus*. Parameters inputs are in Table A. Diet data were compiled as for Node 2.

**Table A. Copepods groups data and data sources.**

| Node | Group name | B (mt.km^-2^) | Data source | P/B (yr^-1^) | Data source | Q/B (yr^-1^) | Data source |
| --- | --- | --- | --- | --- | --- | --- | --- |
| 4 | Copepods S | 7.81 | EMAX initial inputs | 42.57 | EMAX balanced outputs | 127.75 | EMAX balanced outputs |
| 5 | Copepods L | 7.63 | EMAX balanced outputs | 48.52 | EMAX initial inputs | 109.50 | EMAX balanced outputs |

**Node 6. Gelatinous zooplankton**

The gelatinous zooplankton functional group includes cnidarians, ctenophores, colonial siphonophores, and salps. Estimates were obtained from the weighted average of EMAX balanced outputs. Biomass was 1.013 mt.km^-2^, P/B was 37.967 yr^-1^, and Q/B was 145.326 yr^-1^. Diet data were compiled as described for Node 2.

**Node 7. Micronekton**

The micronekton group is made up of large-bodied plankton falling within, or larger than the 5-10mm range, including Amphipoda, Euphausiacea, Mysidacea, Decapoda, and Chaetognatha. Biomass (7.65 mt.km^-2^) and Q/B (85.49 yr^-1^) parameters were derived from EMAX balanced outputs, while P/Q (14.25 yr^-1^) came from raw inputs [1]. Diet data was compiled as for Node 2.

**Nodes 8 to 11. Macrobenthos (polychaete, crustaceans, mollusks, and others)**

Macrobenthos groups were created according to Link *et al.* [1]. Input parameters and sources are described in Table B, and Table C lists the taxa in each subgroup. Diet data was compiled as for Node 2.

**Table B. Macrobenthos groups data and data sources.**

| Node | Group name | B (mt.km^-2^) | Data source | P/B (yr^-1^) | Data source | Q/B (yr^-1^) | Data source |
| --- | --- | --- | --- | --- | --- | --- | --- |
| 8 | Macrobenthos polychaete | 14.68 | Estimate started from EMAX initial inputs | 2.51 | EMAX balanced outputs | 17.5 | EMAX balanced outputs |
| 9 | Macrobenthos crustaceans | 5.89 | EMAX initial inputs | 3.06 | EMAX balanced outputs | 21 | EMAX balanced outputs |
| 10 | Macrobenthos mollusks | 8.34 | EMAX initial inputs | 2.04 | EMAX balanced outputs | 13.95 | EMAX balanced outputs |
| 11 | Macrobenthos others | 8.89 | Estimate started from EMAX initial inputs | 2.02 | EMAX balanced outputs | 16.06 | EMAX balanced outputs |

**Table C. Macrobenthos groups and respective lists of taxa.**

| **Macrobenthos polychaete** | **Macrobenthos crustaceans** | **Macrobenthos mollusks** | **Macrobenthos others** |
| --- | --- | --- | --- |
| *Aglaophamus circinata* | *Ampelisca agassizi* | *Anomia aculeata* | *Actinauge verrilli* |
| *Ampharete arctica* | *Ampelisca vadorum* | *Aporrhais occidentalis* | *Actiniaria unident.* |
| *Anobothrus gracilis* | *Amphipoda unident.* | *Astarte crenata* | *Amphioplus abditus* |
| *Aphrodita hastata* | *Byblis serrata* | *Astarte undata* | *Anthozoa* |
| *Aphrodita* sp. | *Cirolana polita* | *Bivalvia unident.* | *Arachnida* |
| *Asabellides oculata* | *Cirolana* sp. | *Buccinum* sp*.* | *Arbacia punctulata* |
| *Capitellidae* | *Corophium crassicorne* | *Buccinum undatum* | *Arbacia punctulata* |
| *Chone infundibuliformis* | *Crangon septemspinosa* | *Busycon canaliculatum* | *Ascidiacea* |
| *Filograna implexa* | *Diastylis quadrispinosa* | *Colus pubescens* | *Asteroidea* |
| *Glycera dibranchiata* | *Edotea acuta* | *Colus pygmaeus* | *Asteroidea* |
| *Lumbrineris acicularum* | *Gammarus annulatus* | *Colus stimpsoni* | *Brisaster fragilis* |
| *Lumbrineris hebes* | *Isopoda unident.* | *Cyclocardia borealis* | *Ceriantheopsis americanus* |
| *Lumbrineris magalhaensis* | *Leptocheirus pinguis* | *Ensis directus* | *Cerianthus* sp*.* |
| *Lumbrineris* sp*.* | *Meganyctiphanes* sp. | *Euspira heros* | *Craniella* |
| *Maldane sarsi* | *Pagurus acadianus* | *Gastropoda unident.* | *Cucumariidae unident.* |
| *Nephtys bucera* | *Pagurus longicarpus* | *Lunatia triseriata* | *Echinarachnius parma* |
| *Nephtys incisa* | *Pagurus pollicaris* | *Modiolus modiolus* | *Echinarachnius parma* |
| *Nephtys picta* | *Pagurus pubescens* | *Mytilus edulis* | *Edwardsia elegans* |
| *Nephtys* sp*.* | *Pagurus* sp*.* | *Nassarius trivittatus* | *Encope emarginata* |
| *Ninoe nigripes* | *Politolana impressa* | *Neverita duplicata* | *Havelockia scabra* |
| *Onuphis opalina* | *Politolana polita* | *Nucula proxima* | *Hydrozoa* |
| *Pherusa affinis* | *Pseudunciola obliqua* | *Nucula* sp. | *Hydrozoa* |
| *Polychaeta unident.* | *Rhepoxynius hudsoni* | *Pitar morrhuanus* | *Mellita quinquiesperforata* |
| *Scalibregma inflatum* | *Unciola inermis* | *Pleurobranchaea* sp. | *Molgula arenata* |
| *Spio filicornis* | *Unciola irrorata* | *Pleurobranchaea tarda* | *Molpadia oolitica* |
| *Spio setosa* | *Unciola* sp*.* |  | *Ophiura sarsi* |
| *Spiophanes bombyx* |  |  | *Ophiuroidea unident.* |
| *Sternaspis fossor* |  |  | *Paranthus rapiformis* |
| *Sternaspis scutata* |  |  | *Pennatula aculeata* |
| *Streblosoma* sp*.* |  |  | *Porifera* |
|  |  |  | *Rhynchocoela* |
|  |  |  | *Schizaster* sp. |
|  |  |  | *Sclerodactyla briareus* |
|  |  |  | *Steroderma unisemita* |
|  |  |  | *Strongylocentrotus droebachiensis* |

**Nodes 12 and 13. Megabenthos filter feeders and other**

Large-bodied benthic invertebrates fall in the megabenthos category. Megabenthos filter feeders include commercially exploited bivalves (*Artica islandica*, *Mercenaria mercenaria*, *Placopecten magellanicus*, and *Spisula solidissima*). Input parameters were estimated from EMAX models, as previously described. Biomass (2.99 mt.km^-2^) was derived from EMAX inputs [1], using NEFSC clam surveys from 1997 and 1999. P/B (3.93 yr ^-1^) and Q/B (16.51 yr^-1^) were derived from EMAX balanced estimates [2]. The other Megabenthos group includes sea stars and large arthropods, such as horseshoe crabs and lobsters (Table D). Biomass (4.49 mt.km^-2^) was estimated from the initial EMAX biomass input (NEFSC bottom trawl survey), P/B (1.89 yr^-1^) was calculated from EMAX balanced outputs, Q/B (9.53 yr^-1^) started as the EMAX Gulf of Maine model estimate but was modified during the balancing process. Diet data were compiled as for Node 2.

**Table D. Megabenthos-other list of family, genus, and species.**

| Megabenthos other | | |
| --- | --- | --- |
| *Asterias forbesii* | *Galatheidae* | *Octopus vulgaris* |
| *Asterias vulgaris* | *Geryon affinis* | *Ovalipes ocellatus* |
| *Astropecten* spp. | *Geryon fenneri* | *Ovalipes stephensoni* |
| *Calappa flammea* | *Geryon quinquedens* | *Paguristes erythrops* |
| *Calappa sulcata* | *Hepatus epheliticus* | *Panulirus argus* |
| *Callinectes sapidus* | *Homarus americanus* | *Portunus gibbesii* |
| *Callinectes similis* | *Leptasterias* sp. | *Scyllarides aequinoctialis* |
| *Cancer borealis* | *Libinia dubia* | *Scyllarides nodifer* |
| *Cancer irroratus* | *Libinia emarginata* | *Solaster* sp*.* |
| *Carcinus maenas* | *Limulus polyphemus* | *Squilla* |
| *Chionoecetes opilio* | *Lithodes maja* | *Stomatopoda* |

**Node 14. Shrimp**

This functional group includes a variety of species (*Farfantepenaeus aztecus, Farfantepenaeus duorarum, Litopenaeus setiferus, Pandalus borealis, Pandalus* spp*., Pandalus montagui, Pandalus propinquus, Pasiphaea multidentate, Rimapenaeus constrictus*). The initial biomass estimate from the EMAX biomass input increased during the balancing process to a final biomass of 1.96 mt.km^-2^. Uncertainty in the EMAX estimates prompted us to allow extra room for changes. For P/B (1 yr^-1^) we used a weighted average of initial EMAX input data, and employed the EMAX Q/B (5 yr^-1^) input. Diet data was compiled as described for Node 2.

**Node 15. Mesopelagic**

The group includes lanternfishes (*Myctophidae*) and marine hatchefishes (*Maurolicus* sp.). For biomass (0.15mt.km^-2^) and P/B (0.65 yr^-1^) we started with EMAX output values, which were changed slightly during the balancing process [2]. The EMAX output for Q/B (1.82 yr^-1^) remained the same for this group. Diet data was compiled as described for Node 2.

**Node 16. Atlantic herring**

Atlantic herring (*Clupea harengus*) support an important fishery in the Northeast US. Over many years, its population complex has undergone steep decline followed by quick recovery [11]. We relied on data from the Atlantic herring stock assessment [12]. Biomass was estimated to be 6.19 mt.km^-2^, and the P/B ratio was 0.62 yr^-1^ [3]. The Q/B (1.97 yr^-1^) was based on EMAX estimates [2]. The NEFSC trawl survey’s food habits program determined diet composition [3].

**Node 17. Anadromous alosine**

The anadromous alosine functional group encompasses American shad (*Alosa sapidissima*), as well as blueback herring (*A. aestivalis*) and alewife (*A. pseudoharengus*) (together river herring). Biomass (0.076 mt.km^-2^) for anadromous alosine group was based on NEFSC survey estimates, with catchability corrected for the 2000 year-block. The EMAX model input value for P/B (0.44 yr^-1^) was changed during the balancing process, due to the high group mortality, resulting in as estimate of 1.3 yr^-1^. The Q/B ratio, 9.4 yr^-1^, averaged herring and alewife consumption ratios for the EMAX Chesapeake Bay model [13]. Diet composition integrated and averaged fish food habit data from NEFSC and NEAMAP surveys [3].

**Nodes 18 to 20. Atlantic menhaden S, M and L**

Atlantic menhaden (*Brevoortia tyrannus*) are part of the forage fish complex. Copious information about this valuable, heavily fished species allowed us to separate them in different age/size classes according to Buchheister *et al.* [3] (S = age 0, ≤ 14 cm; M = age 1-2, 15-24 cm; L = age 3+, >24). Stock assessments provided biomass estimates for the individual size classes [14], each of which was treated as a different functional group to simplify analysis. Biomasses for the small, medium and large Atlantic menhaden groups were 1.58, 2.87 and 0.48 mt.km^-2^, respectively. For the large class, our initial stock assessment estimate of 0.43 mt.km^-2^ increased by 0.05 mt.km^-2^ during balancing.

The P/B ratios are given at the population level. Therefore, we calculated P/B for each age class using Gascuel *et al.* [15], who recommended using their empirical equation in the absence of data. The P/B ratios for small, medium and large groups were 1.5, 0.93 and 0.90 yr^-1^, respectively. Table E shows the P/B values calculated for small, medium and large menhaden as well as for other species separated into functional groups by age class.

The Q/B ratio was set as 15.86 yr^-1^ for the small Atlantic menhaden functional group [13]. For medium (7.0 yr^-1^) and large (4.3 yr^-1^) groups, Q/B ratios came from multistanza calculations based on Christensen *et al.* [13]. Diets followed the approach in Buchheister *et al.*[3].

**Table E. Parameters for production (P/B) calculation and references.**

| **Functional groups** | **K** | **Reference** | **Trophic level Ʈ** | **Θ (^o^C)** | **Reference** | **P/B (yr^-1^)** |
| --- | --- | --- | --- | --- | --- | --- |
| Atlantic menhaden (S) | 0.42 | [13] | 2.39 | 18 | [16] | 1.019 |
| Atlantic menhaden (M) | 0.42 | [13] | 2.70 | 18 | [16] | 0.925 |
| Atlantic menhaden (L) | 0.42 | [13] | 2.79 | 18 | [16] | 0.903 |
| Bluefish (S) | 0.26 | [13,17] | 4.41 | 24.8 | [18] | 0.514 |
| Bluefish (M) | 0.26 | [13,17] | 4.45 | 24.8 | [18] | 0.510 |
| Bluefish (L) | 0.26 | [13,17] | 4.66 | 24.8 | [18] | 0.492 |
| Striped bass (S) | 0.11 | [13] | 4.19 | 15 | [19,20] | 0.240 |
| Striped bass (M) | 0.11 | [13] | 4.04 | 15 | [19,20] | 0.247 |
| Striped bass (L) | 0.11 | [13] | 4.21 | 15 | [19,20] | 0.240 |
| Weakfish (S) | 0.26 | [13] | 4.07 | 15 | [19–21] | 0.449 |
| Weakfish (M) | 0.26 | [13] | 4.31 | 15 | [19–21] | 0.430 |
| Weakfish (L) | 0.26 | [13] | 4.39 | 15 | [19–21] | 0.423 |
| Dogfish (S) | 0.11 | [22] | 4.05 | 15 | [19,20] | 0.247 |
| Dogfish (L) | 0.11 | [22] | 4.11 | 15 | [19,20] | 0.244 |
| Atlantic cod (S) | 0.28 | [23] | 3.71 | 12 | [19,24] | 0.479 |
| Atlantic cod (M) | 0.28 | [23] | 3.92 | 12 | [19,24] | 0.459Ch |
| Atlantic cod (L) | 0.28 | [23] | 4.21 | 12 | [19,24] | 0.434 |
| Yellowtail flounder (S) | 0.90 | SNE MAB [12] | 3.59 | 10 | [19,25] | 1.073 |
| Yellowtail flounder (L) | 0.90 | SNE MAB [12] | 3.49 | 10 | [19,25] | 1.099 |
| Summer flounder (S) | 0.29 | [3] | 4.24 | 23 | [19,26] | 0.563 |
| Summer flounder (L) | 0.29 | [3] | 4.54 | 23 | [19,26] | 0.534 |

**Node 21. Anchovies**

The anchovies functional group includes bay anchovy (*Anchoa mitchilli*), silver anchovy (*Engraulis eurystole*), and striped anchovy (*A. hepsetus*). An initial biomass estimate based on NEFSC trawl surveys was changed during the balancing process, resulting in a final estimate of 2.31 mt.km^-2^. We adopted Christensen and colleagues’ [13] parameters for bay anchovy for this functional group: P/B (3 yr^-1^) and Q/B (10.9 yr^-1^), and estimated diet using NEFSC survey data.

**Node 22. Atlantic mackerel**

Atlantic mackerel (*Scomber scombrus*) is a small schooling pelagic. Considered to be a forage fish today, it once supported a valuable fishery. We obtained the parameters for this functional group from the NEFSC surveys and EMAX models. The biomass estimate was 0.77 mt.km^-2^, using NEFSC trawl surveys with corrected catchability. The P/B (0.38 yr^-1^) and Q/B (1.97 yr^-1^) ratios were derived from the EMAX models’ commercial small pelagics functional group, and diets were estimated from NEFSC survey data.

**Node 23. Squid**

The squid functional group is composed of longfin inshore squid (*Doryteuthis pealeii*) and northern shortfin squid (*Illex illecebrosus*). Biomass (1.06 mt.km^-2^) was estimated from NEFSC survey data, while P/B (0.97 yr^-1^), Q/B (2.70 yr^-1^) and diet estimates came from the EMAX models, using the approach described in Node 2.

**Node 24. Butterfish**

The butterfish (*Peprilus triacanthus*) functional group parameter estimates were derived from various sources. The final biomass of 0.89 mt.km^-2^ was based on initial input from the NEFSC trawl surveys (0.80 mt.km^-2^). P/B (1.27 yr^-1^) was obtained from stock assessments [27], while the Q/B ratio (1.97 yr^-1^) was calculated from EMAX models using methods described in Node 2. Diets were computed from NEFSC food habits data.

**Node 25. Small pelagics**

The small pelagics functional group includes all other forage fishes with too little resolution to form a functional group (Table F). Our initial biomass estimate, based on NEFSC survey data (0.24 mt.km^-2^), changed during balancing to result in a final estimate of 0.29 mt.km^-2^. The initial P/B estimate, based on EMAX model estimates (0.82 yr^-1^), changed during the balancing process to a final estimate of 0.97 yr^-1^. The Q/B ratio (4 yr^-1^) was based on the Christensen *et al.*[13] littoral forage fish group. Diet data were obtained from the EMAX models following the methods described in Node 2.

**Table F. List of species included in the small pelagic functional group.**

| **Small pelagics** | |
| --- | --- |
| *Ablennes hians* | *Opisthonema oglinum* |
| *Ammodytes americanus* | *Osmerus mordax* |
| *Ammodytes dubius* | *Peprilus alepidotus* |
| *Argentina silus* | *Sardinella aurita* |
| *Ariomma bondi* | *Scomber japonicus* |
| *Decapterus macarellus* | *Scomberesox saurus* |
| *Decapterus punctatus* | *Scomberomorus cavalla* |
| *Etrumeus teres* | *Scomberomorus maculatus* |
| *Menidia menidia* | *Selar crumenophthalmus* |
| *Mugil cephalus* | *Selene setapinnis* |
| *Mugil curema* | *Trachurus lathami* |

**Nodes 26 to 28. Bluefish S, M and L**

To account for age-related dietary shifts, bluefish (*Pomatomus saltatrix*) were divided in three age/size classes (S = age 0, < 30 cm; M = age 1-3, 30 - 60 cm; L = age 4+, > 60 cm) according to methodology in Buchheister *et al.* [3]. We treated each age class as a different functional group. Biomasses obtained from NEFSC stock assessments [14] for the small, medium and large bluefish groups were 0.04, 0.05 and 0.19 mt.km^-2^, respectively, for year block 2000.

Since P/B ratios are given at the population level, we calculated P/B for each age class using Gascuel *et al.* [15]. For small, medium and large groups, P/B ratios were 0.51, 0.51 and 0.49 yr^-1^ respectively (Table E).

The Q/B ratios were set as 18.11 yr^-1^ for the small bluefish functional group [13]. For medium (3.52 yr^-1^) and large (1.93 yr^-1^) groups, Q/B ratios were obtained from multistanza calculations based on Christensen *et al.* [13]. Diets followed Buchheister *et al.*[3].

**Nodes 29 to 31. Striped bass S, M and L**

Striped bass (*Morone saxatilis*) was divided in three age/size classes (S = age 0-1, ≤ 25 cm; M = age 2-6, 26 - 70 cm; L = age 7+, > 70 cm) to account for ontogenetic shifts using Buchheister *et al.* [3] methodology. We treated each age class as a functional group, and obtained its biomass from stock assessments [14]. Biomass for small, medium and large striped bass groups were 0.06, 0.36 and 0.28 mt.km^-2^, respectively [28].

Using Gascuel *et al.* [15], P/B ratios for each age class were calculated: the small, medium and large groups were 0.25, 0.24 and 0.24 yr^-1^, respectively (Table E). The Q/B ratio was set at 23.27 yr^-1^ for the small striped bass functional group [13]. For medium (6.34 yr^-1^) and large (3.19 yr^-1^) groups, we obtained Q/B ratios using multistanza calculations based on Christensen *et al.* [13]. Diets followed methods in Buchheister *et al.*[3].

**Nodes 32 to 34. Weakfish S, M and L**

Weakfish (*Cynoscion regalis*) were divided in three age/size classes (S = age 0, ≤ 20 cm; M = age 1-2, 21 - 40 cm; and L = age 3+, > 40 cm), which we treated as functional groups based on diet changes using methodology in Buchheister *et al.* [3]. Biomasses came from NEFSC surveys. For small, medium and large striped bass groups, they were 0.15, 0.30 and 0.036 mt.km^-2^, respectively.

P/B ratios calculated for the small, medium and large age class groups using Gascuel *et al.* [15] were 0.44, 0.43 and 0.42 yr^-1^, respectively (Table E). The Q/B ratio was set as 13.52 yr^-1^ for the small weakfish functional group [13]. Multistanza calculations based on Christensen *et al.* [13] yielded Q/B ratios for the medium (4.21 yr^-1^) and large (2.44 yr^-1^). Diets followed Buchheister *et al.*[3].

**Nodes 35 to 36. Dogfish S and L**

Spiny dogfish (*Squalus acanthias*) were separated in two age/size groups (S = age 0-5, < 60 cm; and L = age 6+, > 60 cm) based on methods in Buchheister *et al.* [3] that consider changes in diet. We analyzed these age/size classes as functional groups. Biomasses obtained from NEFSC survey were 1.0 and 13.28 mt.km-2, respectively. However, employing the 41 % catchability correction in Sagarese *et al.* [29] resulted in values of 0.41 and 5.44 mt.km-2. Biomasses changed further during balancing, yielding final estimates of 0.47 and 2.7 mt.km^-2^ for small and large spiny dogfish, respectively.

Calculated using Gascuel *et al.* [15], the P/B ratio for both functional groups was 2.4 yr^-1^ (Table E). The Q/B ratios were set at 1.47 yr^-1^ for the small dogfish functional group, and 0.60 yr^-1^ for the large dogfish functional group. Initial Q/B inputs based on Christensen *et al.* [13] and Araujo and Bundy [30] changed during balancing. Diets followed Buchheister *et al.*[3].

**Nodes 37 to 39. Atlantic cod S, M and L**

We divided the Atlantic cod (*Gadus morhua*) functional group into three age/size categories to account for ontogenetic shifts (S = age 0-1, ≤ 20 cm; M = age 2 – 3, 21 – 50 cm; and L = age 4+, > 50 cm) based on Buchheister *et al.* [3], and treated these categories as functional groups in our models. Initial biomasses (0.01, 0.08, and 0.08 mt.km^-2^) were obtained from the Gulf of Maine and Georges Bank stock assessment reports [23]. For the small cod group, the initial estimate changed to a final biomass of 0.02 mt.km^-2^.

The P/B ratios were calculated for each age/size class using Gascuel *et al.* [15]. Results for small, medium and large groups were 0.47, 0.45, and 0.43 yr^-1^, respectively (Table E). For large cod, the Q/B ratio was set to 2.2 yr^-1^ based on Georges Bank estimate found in Pauly [31]. For medium (3.4 yr^-1^) and small (6.9 yr^-1^) groups, Q/B ratios were obtained via multistanza calculations. Diets followed the approach in Buchheister *et al.*[3].

**Node 40. Haddock**

The recognized importance of commercial fisheries for haddock (*Melanogrammus aeglefinus*) stocks in Gulf of Maine [32] and Georges Bank[33] motivated us to treat the species as one group. Biomass was set at 0.60 mt.km^-2^, calculated by summing NEFSC survey estimates for the two stocks, and dividing by the combined area, and accounting for corrected catchability.

The P/B ratio was 0.45 yr^-1^ using balanced EMAX model output according to the method described in Node 2. We applied a Q/B ratio of 3 yr^-1^, estimate for Georges Bank [31]. Diets were computed from NEFSC food habits data.

**Node 41. Hakes**

The hake functional group includes species from the genera *Merluccius* (*M. albidus* and *M. bilinearis*) and *Urophycis* (*U. chuss*, *U. regia*, *U. tenuis*). Biomass was estimated to be 0.827 mt.km^-2^ based on NEFSC trawl surveys. The P/Q ratio of 1.11 yr^-1^ [3] came from total mortality estimates for *M. bilinearis*, and the Q/B ratio was 3.85 yr^-1^ [31]. Diets were computed from NEFSC food habits data.

**Node 42. Atlantic croaker**

Atlantic croaker (*Micropogonias undulatus*) biomass was estimated at 0.81 mt.km-^2^ from the stock assessment [34]. We used the EMAX models’ demersal benthivore P/B (0.45 yr^-1^) and Q/B (0.90 yr^-1^) ratios [1]. Diet data averaged NEMAP and NEFSC food habits data.

**Nodes 43 to 44. Yellowtail flounder S and L**

Yellowtail flounder (*Limanda ferruginea*) were divided into two age/size groups (S = age 0, ≤ 20cm; and L= age1+ , > 20 cm). The biomass estimate of 0.043 mt.km^-2^ for the small functional group was calculated using the EwE multistanza group approach, while the 0.11 mt.km^-2^ biomass estimate for the large functional group was based on NEFSC surveys.

We calculated P/B ratios for each age class using Gascuel *et al.* [15], with ratios for small and large groups of 1.07 and 1.09 yr^-1^, respectively (Table E). The Q/B ratios were set at 4.4 yr^-1^ for the small age group, and 2.9 yr^-1^ for the large age group, using the multistanza approach in Christensen *et al.* [13] for summer flounder. Diets followed Buchheister *et al.*[3], informed by the NEFSC survey food habits program.

**Nodes 45 to 46. Summer flounder S and L**

Summer flounder (*Paralichthys dentatus*) were divided into two age/size groups (S = age 0, ≤ 25cm; and L= age1+ , > 25 cm). Biomasses estimates of 0.029 mt.km^-2^ for the small functional group, and 0.17 mt.km^-2^ for the large functional group, were based on the stock assessment [28].

The P/B ratios for each age class, using Gascuel *et al.* [15], were 0.56 and 0.53 yr^-1^ for small and large groups, respectively (Table E). Q/B ratios were set to 4.4 yr^-1^ for the small age group and 2.9 yr^-1^ for the large, using Christensen *et al.* [13] and a multistanza approach. Diets followed Buchheister *et al.*[3], informed by the NEFSC and NEAMAP food habits programs.

**Nodes 47. Skates**

The skates functional group includes the following species *Amblyraja radiata*, *Dipturus laevis*, *Leucoraja erinacea*, *L. garmani*, *L. oceallata*, and *Raja eglanteria*. A biomass of 1.65 mt.km^-2^, derived from NEFSC surveys, was corrected for catchability. The P/B ratio of 0.45 yr^-1^ was estimated using the weighted average of the EMAX model estimates. The Q/B ratio, 2.4 yr^-1^, was within the range of the EMAX models’ demersal functional group [1].

**Node 48. Demersal benthivores**

Remaining demersal species were grouped according to feeding niche: benthivores, piscivores and omnivores. The list of demersal species appears in Table G. The initial biomass input was based on NEFSC surveys (1.74 mt.km^-2^), however changes during the balancing process resulted in a final biomass of 2.05 mt.km^-2^. The P/B (0.45 yr^-1^) and Q/B (0.90 yr^-1^) ratios were generated from the EMAX models’ balanced outputs using the methodology described in Node 2. Diets were calculated from NEFSC and NEAMAP food habits data.

**Table G. List of species included in the demersal benthivore functional group.**

| **Demersal benthivore**s | | | |
| --- | --- | --- | --- |
| *Acipenser oxyrinchus* | *Enchelyopus cimbrius* | *Lycenchelys verrillii* | *Pogonias cromis* |
| *Alectis ciliaris* | *Epigonus pandionis* | *Lycodes reticulatus* | *Polymetme thaeocoryla* |
| *Anarhichas lupus* | *Etmopterus princeps* | *Macrorhamphosus scolopax* | *Polymixia lowei* |
| *Ancylopsetta ommata* | *Etropus crossotus* | *Macrourus berglax* | *Polymixia nobilis* |
| *Antigonia capros* | *Etropus microstomus* | *Macrozoarces americanus* | *Pontinus longispinis* |
| *Antimora rostrata* | *Eucinostomus argenteus* | *Malacocephalus occidentalis* | *Porichthys plectrodon* |
| *Archosargus probatocephalus* | *Eumicrotremus spinosus* | *Malacoraja senta* | *Prionotus carolinus* |
| *Artediellus* sp. | *Gaidropsarus ensis* | *Menticirrhus saxatilis* | *Prionotus evolans* |
| *Aspidophoroides monopterygius* | *Gephyroberyx darwini* | *Monolene sessilicauda* | *Prionotus paralatus* |
| *Astroscopus guttatus* | *Glyptocephalus cynoglossus* | *Morone americana* | *Pseudopleuronectes americanus* |
| *Bagre marinus* | *Gobiosoma bosc* | *Mullus auratus* | *Rhinoptera bonasus* |
| *Bairdiella chrysoura* | *Gonostoma atlanticum* | *Mustelus canis* | *Saurida brasiliensis* |
| *Balistes capriscus* | *Gonostoma bathyphilum* | *Myliobatis freminvillei* | *Scophthalmus aquosus* |
| *Bothus ocellatus* | *Gonostoma elongatum* | *Myoxocephalus aenaeus* | *Sebastes fasciatus* |
| *Brosme brosme* | *Gymnachirus melas* | *Myoxocephalus octodecemspinosus* | *Selene vomer* |
| *Caranx hippos* | *Helicolenus dactylopterus* | *Myoxocephalus scorpius* | *Sphoeroides maculatus* |
| *Careproctus ranula* | *Helicolenus maderensis* | *Nesiarchus nasutus* | *Stenotomus chrysops* |
| *Chauliodus danae* | *Hemitripterus americanus* | *Nezumia bairdi* | *Synagrops bellus* |
| *Chilomycterus schoepfii* | *Hippoglossina oblonga* | *Ogcocephalus corniger* | *Synagrops spinosus* |
| *Chlorophthalmus agassizi* | *Hippoglossoides platessoides* | *Ophidion grayi* | *Tautoga onitis* |
| *Chlorophthalmus* sp. | *Hoplostethus occidentalis* | *Ophidion marginatum* | *Tautogolabrus adspersus* |
| *Chloroscombrus chrysurus* | *Howella sherborni* | *Ophidion welshi* | *Torpedo nobiliana* |
| *Citharichthys arctifrons* | *Lagodon rhomboides* | *Opsanus pardus* | *Trachinotus carolinus* |
| *Coelorhynchus carminatus* | *Larimus fasciatus* | *Opsanus tau* | *Trachinotus falcatus* |
| *Cryptacanthodes maculatus* | *Leiostomus xanthurus* | *Orthopristis chrysoptera* | *Triglops murrayi* |
| *Dasyatis americana* | *Lepophidium profundorum* | *Otophidium omostigmum* | *Trinectes maculatus* |
| *Dasyatis centroura* | *Liparis inquilinus* | *Paralichthys oblongus* | *Ulvaria subbifurcata* |
| *Dasyatis sabina* | *Lopholatilus chamaeleonticeps* | *Parasudis truculenta* | *Vinciguerria sp.* |
| *Dasyatis say* | *Lumpenus lumpretaeformis* | *Peristedion miniatum* | *Xenodermichthys copei* |
| *Dibranchus atlanticus* | *Lumpenus maculatus* | *Poecilopsetta beani* | *Zoarces americanus* |

**Node 49. Demersal piscivores**

Demersal piscivore biomass input was based on NEFSC surveys (0.54 mt.km^-2^). The P/B (0.5 yr^-1^) and Q/B (1.2 yr^-1^) ratios came from the balanced outputs of EMAX models using methods described in Node 2. Diets were calculated from NEFSC and NEAMAP food habits data. A list of demersal piscivores can be found in Table H.

**Table H. List of species included in the demersal piscivore functional group.**

| **Demersal piscivores** | | |
| --- | --- | --- |
| *Centroscyllium fabricii* | *Lutjanus apodus* | *Pollachius virens* |
| *Conger oceanicus* | *Lutjanus buccanella* | *Reinhardtius hippoglossoides* |
| *Gymnura altavela* | *Lutjanus campechanus* | *Scyliorhinus retifer* |
| *Gymnura micrura* | *Lutjanus griseus* | *Squatina dumeril* |
| *Hippoglossus hippoglossus* | *Lutjanus jocu* | *Synodus foetens* |
| *Lophius americanus* | *Lutjanus vivanus* | *Trichiurus lepturus* |
| *Lutjanus analis* | *Myxine glutinosa* | *Urophycis chesteri* |

**Node 50. Demersal omnivores**

The biomass of demersal omnivores was based on NEFSC surveys (0.018 mt.km^-2^). However initial biomass for the node increased during the balancing process––the NEFSC survey estimate is an order of magnitude lower than the landings for some species in the functional group [3]. The P/B (0.45 yr^-1^) and Q/B (0.81 yr^-1^) ratios were generated from the EMAX models’ balanced outputs, with the methodology described in Node 2. Diets were calculated from NEFSC and NEAMAP food habits data. A list of species of demersal piscivores can be found in Table I.

**Table I. List of species included in the demersal omnivore functional group.**

| **Demersal omnivores** | | |
| --- | --- | --- |
| *Abudefduf saxatilis* | *Diplectrum formosum* | *Opistognathus lonchurus* |
| *Acanthurus bahianus* | *Diplodus argenteus* | *Opistognathus maxillosus* |
| *Acanthurus chirurgus* | *Diplodus holbrooki* | *Pagrus sedecim* |
| *Acanthurus coeruleus* | *Dipturus olseni* | *Parablennius marmoreus* |
| *Aluterus heudelotii* | *Dormitator maculatus* | *Parahollardia lineata* |
| *Aluterus monoceros* | *Echeneis naucrates* | *Paralichthys albigutta* |
| *Aluterus schoepfi* | *Echiophis punctifer* | *Paralichthys lethostigma* |
| *Aluterus scriptus* | *Engyophrys senta* | *Paralichthys sp.* |
| *Anchoa lyolepis* | *Epinephelus adscensionis* | *Paralichthys squamilentus* |
| *Ancylopsetta dilecta* | *Epinephelus drummondhayi* | *Paranthias furcifer* |
| *Ancylopsetta quadrocellata* | *Epinephelus flavolimbatus* | *Peristedion gracile* |
| *Anguilla rostrata* | *Epinephelus guttatus* | *Pholis fasciata* |
| *Anisotremus virginicus* | *Epinephelus inermis* | *Pleuronectidae* |
| *Apogon aurolineatus* | *Epinephelus morio* | *Pleuronectiformes* |
| *Apogon maculatus* | *Epinephelus mystacinus* | *Polydactylus octonemus* |
| *Apogon pseudomaculatus* | *Epinephelus nigritus* | *Pomacanthus arcuatus* |
| *Argentina striata* | *Epinephelus niveatus* | *Pomacentrus leucostictus* |
| *Ariomma melanum* | *Epinephelus striatus* | *Pomacentrus variabilis* |
| *Ariomma regulus* | *Equetus acuminatus* | *Pontinus rathbuni* |
| *Astroscopus y-graecum* | *Equetus lanceolatus* | *Priacanthus arenatus* |
| *Balistes vetula* | *Equetus punctatus* | *Priacanthus cruentatus* |
| *Bathygobius soporator* | *Equetus umbrosus* | *Prionotus alatus* |
| *Bathyraja spinicauda* | *Etheostoma nigrum* | *Prionotus longispinosus* |
| *Bellator brachychir* | *Etmopterus gracilispinis* | *Prionotus ophryas* |
| *Bellator egretta* | *Etmopterus hillianus* | *Prionotus roseus* |
| *Bellator militaris* | *Etropus rimosus* | *Prionotus rubio* |
| *Bembrops gobioides* | *Eucinostomus gula* | *Prionotus scitulus* |
| *Bodianus pulchellus* | *Fistularia tabacaria* | *Prionotus stearnsi* |
| *Bothus lunatus* | *Foetorepus agassizi* | *Prionotus tribulus* |
| *Bothus robinsi* | *Gastropsetta frontalis* | *Pristigenys alta* |
| *Brama brama* | *Gempylus serpens* | *Pristipomoides aquilonaris* |
| *Breviraja plutonia* | *Gobiesox strumosus* | *Pseudupeneus maculatus* |
| *Calamus bajonado* | *Gymnothorax saxicola* | *Raja ackleyi* |
| *Calamus calamus* | *Haemulidae* | *Raja texana* |
| *Calamus leucosteus* | *Haemulon aurolineatum* | *Rhomboplites aurorubens* |
| *Calamus nodosus* | *Haemulon carbonarium* | *Ruvettus pretiosus* |
| *Calamus penna* | *Haemulon plumieri* | *Rypticus bistrispinus* |
| *Calamus proridens* | *Haemulon striatum* | *Rypticus subbifrenatus* |
| *Canthidermis sufflamen* | *Halichoeres bathyphilus* | *Scarus coeruleus* |
| *Canthigaster rostrata* | *Halichoeres bivittatus* | *Schultzea beta* |
| *Caranx crysos* | *Halichoeres caudalis* | *Sciaenops ocellatus* |
| *Carcharhinus altimus* | *Halichoeres poeyi* | *Scorpaena agassizi* |
| *Carcharhinus isodon* | *Halichoeres radiatus* | *Scorpaena brasiliensis* |
| *Carcharhinus longimanus* | *Harengula jaguana* | *Scorpaena calcarata* |
| *Carcharhinus perezii* | *Hemanthias aureorubens* | *Scorpaena dispar* |
| *Carcharhinus porosus* | *Hemanthias vivanus* | *Scorpaena grandicornis* |
| *Carcharhinus signatus* | *Hemipteronotus novacula* | *Scorpaena plumieri* |
| *Caulolatilus chrysops* | *Hippocampus erectus* | *Seriola fasciata* |
| *Caulolatilus cyanops* | *Holacanthus bermudensis* | *Seriola zonata* |
| *Caulolatilus intermedius* | *Holacanthus ciliaris* | *Serraniculus pumilio* |
| *Caulolatilus microps* | *Holacanthus tricolor* | *Serranus annularis* |
| *Centropristis ocyurus* | *Holanthias martinicensis* | *Serranus atrobranchus* |
| *Centropristis philadelphica* | *Hyperoglyphe perciformis* | *Serranus baldwini* |
| *Centropristis striata* | *Hypleurochilus geminatus* | *Serranus notospilus* |
| *Centroscymnus coelolepis* | *Hypsoblennius hentz* | *Serranus phoebe* |
| *Chaetodipterus faber* | *Hypsoblennius ionthas* | *Serranus subligarius* |
| *Chaetodon aculeatus* | *Kathetostoma albigutta* | *Sparisoma radians* |
| *Chaetodon aya* | *Kyphosus sectatrix* | *Sphoeroides dorsalis* |
| *Chaetodon capistratus* | *Lachnolaimus maximus* | *Sphoeroides nephelus* |
| *Chaetodon ocellatus* | *Lactophrys bicaudalis* | *Sphoeroides pachygaster* |
| *Chaetodon sedentarius* | *Lactophrys polygonia* | *Sphoeroides spengleri* |
| *Chaetodon striatus* | *Lactophrys quadricornis* | *Sphoeroides testudineus* |
| *Chaetodontidae* | *Lactophrys trigonus* | *Sphyraena barracuda* |
| *Chascanopsetta lugubris* | *Lactophrys triqueter* | *Sphyraena borealis* |
| *Chasmodes bosquianus* | *Laemonema barbatulum* | *Sphyraena guachancho* |
| *Chaunax stigmaeus* | *Lepidocybium flavobrunneum* | *Sphyrna media* |
| *Chilomycterus antillarum* | *Lobotes surinamensis* | *Stellifer lanceolatus* |
| *Chilomycterus atinga* | *Lutjanus synagris* | *Stenotomus caprinus* |
| *Chromis enchrysurus* | *Macroramphosus scolopax* | *Syacium gunteri* |
| *Chromis insolata* | *Malacanthus plumieri* | *Syacium micrurum* |
| *Citharichthys arenaceus* | *Menticirrhus americanus* | *Syacium papillosum* |
| *Citharichthys cornutus* | *Menticirrhus littoralis* | *Symphurus civitatus* |
| *Citharichthys gymnorhinus* | *Monacanthus ciliatus* | *Symphurus diomedianus* |
| *Citharichthys macrops* | *Mugil gyrans* | *Symphurus marginatus* |
| *Citharichthys* sp. | *Mugil liza* | *Symphurus minor* |
| *Citharichthys spilopterus* | *Mustelus norrisi* | *Symphurus plagiusa* |
| *Clepticus parrae* | *Mycteroperca bonaci* | *Symphurus pusillus* |
| *Cookeolus japonicus* | *Mycteroperca interstitialis* | *Symphurus urospilus* |
| *Cryptotomus roseus* | *Mycteroperca microlepis* | *Syngnathus fuscus* |
| *Cubiceps pauciradiatus* | *Mycteroperca phenax* | *Trachyscorpia cristulata* |
| *Cyclopsetta fimbriata* | *Mycteroperca venenosa* | *Triakis semifasciata* |
| *Cynoscion arenarius* | *Myliobatis goodei* | *Upeneus parvus* |
| *Cynoscion nebulosus* | *Myoxocephalus quadricornis* | *Uranoscopidae* |
| *Cynoscion nothus* | *Narcine brasiliensis* | *Uraspis secunda* |
| *Dactylopterus volitans* | *Neomerinthe hemingwayi* | *Urolophus jamaicensis* |
| *Dasyatis violacea* | *Nicholsina usta* | *Xenocephalus egregius* |
| *Decodon puellaris* | *Ocyurus chrysurus* | *Xenolepidichthys dalgleishi* |
| *Diodon holocanthus* | *Ogcocephalus radiatus* |  |
| *Diodon hystrix* | *Ophidion beani* |  |
| *Diplectrum bivittatum* | *Ophidion selenops* |  |

**Node 51. Medium pelagic**

The medium pelagic functional group includes Atlantic bonito (*Sarda sarda*), cero (*Scomberomous regalis*), and cobia (*Rachycentron canadum*). Biomass for the group was 0.12 mt.km^-2^, based on NEFSC surveys. The P/B (0.45 yr^-1^) and Q/B (1.83 yr^-1^) ratios were generated from the EMAX models’ balanced outputs with methods described in Node 2. Diets were calculated from NEFSC and NEAMAP food habits data.

**Node 52. Coastal sharks**

The coastal sharks group includes Atlantic sharpnose sharks (*Rhizoprionodon terraenovae*), sand tiger sharks (*Carcharias taurus*), dusky sharks (*Carcharhinus obscurus*), sandbar sharks (*C. plumbeus*), and spinner sharks (*C. brevipinna*). Based on outputs from the EMAX models, biomass for this group was 0.01 mt.km^-2^ [2], and we used a Q/B ratio of 1.24 yr^-1^ based on EMAX estimates [1]. However, the initial P/B input (0.1 yr^-1^), also based on the EMAX models, doubled during balancing to 0.2 yr^-1^. Diets derived from the EMAX models accounted for the increased resolution of functional groups.

**Node 53. Pelagic sharks**

The pelagic shark node includes blue sharks (*Prionace glauca*), great white sharks (*Carcharodon carcharias*), hammerheads (*Sphyrna* spp.), makos (*Isurus spp.*), porbeagles (*Lamna lewini*), and thresher sharks (*Alopias vulpinus*). The biomass (0.01 mt.km^-2^), P/B (0.13 yr^-1^), and Q/B (yr^-1^) ratios where obtained from weighted averages of EMAX estimates [1]. Diets derived from EMAX models accounted for increased resolution of the functional groups.

**Node 54. Large pelagics (HMS)**

The large pelagics group represents highly migratory species (HMS) such as tunas (*Thunnus* sp.), billfish (members from Istiophoridae family), and swordfish (*Xiphias gladius*). Our parameters were based on the EMAX models’ highly migratory species node, and modified to exclude coastal and pelagic sharks (Nodes 52 and 53, respectively, in this study). Final biomass output was 0.07 mt.km^-2^, P/B was 0.57 yr^-1^, and Q/B 6.79 yr^-1^[1]. Diets were also based on EMAX models, accounting for the higher resolution of the functional groups [1].

**Node 55. Pinnipeds**

The node representing seals includes harbor seals (*Phoca vitulina*), gray seals (*Halichoerus grypus*), harp seals (*Pagophilus groenlandicus*), and hooded seals (*Cystophora cristata*). The Input parameters were extracted from the EMAX models following methods described for Node 2. Biomass was 0.03 mt.km^-2^, P/B was 0.07 yr^-1^, and Q/B 5.5 yr^-1^ [1]. Diets based on the EMAX models accounted for the higher resolution of functional groups [1].

**Node 56. Baleen whales**

Fin whales (*Balaenoptera physalus*), sei whales (*B. borealis*), minke whales (*B. acutorostrata*), humpback whales (*Megaptera novaeangliae*), and right whales (*Eubalaena glacialis*) form this functional group. Input parameter extracted from the EMAX models follow methods described in Node 2. Biomass was 0.46 mt.km^-2^, P/B was 0.04 yr^-1^, and Q/B 3.21 yr^-1^ [1]. Diets also based on the EMAX models account for higher resolution in functional groups. [1].

**Node 57. Odontocetes**

This functional group includes beaked whales (*Mesoplodon* spp.), bottlenose dolphins (*Tursiops truncatus*), common dolphins (*Delphinus delphis*), dwarf sperm whales (*Kogia* spp.), harbor porpoises (*Phocena phocena*), pilot whales (*Globicephala* spp.), Risso’s dolphins (*Grampus griseus*), sperm whales (*Physeter macrocephalus*), spotted dolphins (*Stenella coeruleoalba*), and white-sided dolphins (*Lagenorhynchus acutus*). Parameters were extracted from the EMAX models following the methodology described in Node 2. Biomass was 0.06 mt.km^-2^, P/B was 0.04 yr^-1^, and Q/B 14.3 yr^-1^ [1]. Diets based on EMAX models accounted for higher resolution in the functional groups [1].

**Node 58. Seabirds**

Seabirds included black-legged kittiwakes (*Rissa triactyla*), Cory’s shearwaters (*Calonectris diomedae*), great black-backed gulls (*Larus marinus*), greater shearwaters (*Puffinus gravis*), herring gulls (*Larus argentatus*), laughing gulls (*Larus philadelphia*), northern fulmars (*Fulmarus glacialis*), and northern gannets (*Sula bassanus*). Parameters extracted from EMAX models follow the methodology described in Node 2. Biomass was 0.007 mt.km^-2^, P/B was 0.27 yr^-1^, and Q/B 9.31 yr^-1^ [1]. Diets based on EMAX models accounting for the higher resolution of functional groups [1].

**Node 59. Detritus**

Detritus accounts for both dissolved and particulate organic carbon (DOC and POC). The parameters for this node were based on the weighted averages of EMAX model estimates. In Ecopath with Ecosim, biomass is the only input estimate for the detritus group, which was 52.6 mt.km^-2^.

The Tables J and K shows the data inputs for calculation of alewife historical biomass data.

**Table J. Alewife abundance time series for New England watersheds based on spawning habitat availability.**

| Year | Total New England spawning habitat (km^2^) | N(YOY) | N+2 | N+3 | N+ 3.583 |
| --- | --- | --- | --- | --- | --- |
| 1600 | 1282 | 2836835448 | 395180952 | 177566248 | 111379553 |
| 1610 | 1282 | 2836835448 | 395180952 | 177566248 | 111379553 |
| 1620 | 1282 | 2836835448 | 395180952 | 177566248 | 111379553 |
| 1630 | 1282 | 2836835448 | 395180952 | 177566248 | 111379553 |
| 1640 | 1233 | 2729260334 | 380195368 | 170832791 | 107155950 |
| 1650 | 1224 | 2708232277 | 377266087 | 169516580 | 106330348 |
| 1660 | 1211 | 2679457041 | 373257597 | 167715449 | 105200578 |
| 1670 | 1189 | 2631424531 | 366566503 | 164708947 | 103314730 |
| 1680 | 1172 | 2593795376 | 361324632 | 162353623 | 101837338 |
| 1690 | 1162 | 2570996534 | 358148675 | 160926573 | 100942212 |
| 1700 | 1143 | 2529825812 | 352413451 | 158349571 | 99325771 |
| 1710 | 1116 | 2470725903 | 344180631 | 154650326 | 97005396 |
| 1720 | 1072 | 2372626696 | 330515073 | 148509995 | 93153835 |
| 1730 | 1067 | 2362666037 | 329127519 | 147886527 | 92762760 |
| 1740 | 1032 | 2283866159 | 318150424 | 142954201 | 89668927 |
| 1750 | 1021 | 2259517882 | 314758626 | 141430167 | 88712968 |
| 1760 | 928 | 2054923074 | 286257864 | 128623949 | 80680187 |
| 1770 | 844 | 1867300344 | 260121371 | 116880066 | 73313762 |
| 1780 | 818 | 1810613128 | 252224647 | 113331839 | 71088114 |
| 1790 | 815 | 1803707071 | 251262609 | 112899568 | 70816969 |
| 1800 | 706 | 1563699465 | 217828723 | 97876754 | 61393814 |
| 1810 | 567 | 1254551607 | 174763361 | 78526240 | 49256081 |
| 1820 | 468 | 1034863745 | 144160085 | 64775302 | 40630718 |
| 1830 | 313 | 692283489 | 96437475 | 43332151 | 27180366 |
| 1840 | 162 | 357494682 | 49800241 | 22376691 | 14035921 |
| 1850 | 152 | 335359885 | 46716787 | 20991206 | 13166867 |
| 1860 | 151 | 334031797 | 46531780 | 20908077 | 13114723 |
| 1870 | 136 | 301692859 | 42026855 | 18883883 | 11845035 |
| 1880 | 118 | 261850224 | 36476638 | 16390010 | 10280738 |
| 1890 | 117 | 258308656 | 35983286 | 16168333 | 10141689 |
| 1900 | 116 | 257201916 | 35829113 | 16099058 | 10098236 |

**Table K. Alewife biomass time series for New England watersheds based on spawning habitat availability.**

| Year | Total New England spawning habitat (km^2^) | N(YOY) W=4.809 | N+2  W=166.66 | N+3 W=199.99 | N+ 3.583 W=204 |
| --- | --- | --- | --- | --- | --- |
| 1600 | 1282 | 13644.9 | 65863.3 | 35512.4 | 22721.4 |
| 1610 | 1282 | 13644.9 | 65863.3 | 35512.4 | 22721.4 |
| 1620 | 1282 | 13644.9 | 65863.3 | 35512.4 | 22721.4 |
| 1630 | 1282 | 13644.9 | 65863.3 | 35512.4 | 22721.4 |
| 1640 | 1233 | 13127.5 | 63365.7 | 34165.8 | 21859.8 |
| 1650 | 1224 | 13026.4 | 62877.5 | 33902.5 | 21691.4 |
| 1660 | 1211 | 12888.0 | 62209.4 | 33542.3 | 21460.9 |
| 1670 | 1189 | 12656.9 | 61094.2 | 32941.0 | 21076.2 |
| 1680 | 1172 | 12475.9 | 60220.6 | 32470.0 | 20774.8 |
| 1690 | 1162 | 12366.3 | 59691.2 | 32184.6 | 20592.2 |
| 1700 | 1143 | 12168.3 | 58735.4 | 31669.2 | 20262.5 |
| 1710 | 1116 | 11884.0 | 57363.2 | 30929.4 | 19789.1 |
| 1720 | 1072 | 11412.1 | 55085.7 | 29701.3 | 19003.4 |
| 1730 | 1067 | 11364.2 | 54854.4 | 29576.6 | 18923.6 |
| 1740 | 1032 | 10985.2 | 53024.9 | 28590.2 | 18292.5 |
| 1750 | 1021 | 10868.1 | 52459.6 | 28285.4 | 18097.4 |
| 1760 | 928 | 9884.0 | 47709.5 | 25724.2 | 16458.8 |
| 1770 | 844 | 8981.6 | 43353.4 | 23375.5 | 14956.0 |
| 1780 | 818 | 8708.9 | 42037.3 | 22665.8 | 14502.0 |
| 1790 | 815 | 8675.7 | 41877.0 | 22579.4 | 14446.7 |
| 1800 | 706 | 7521.3 | 36304.7 | 19574.9 | 12524.3 |
| 1810 | 567 | 6034.3 | 29127.1 | 15704.9 | 10048.2 |
| 1820 | 468 | 4977.6 | 24026.6 | 12954.8 | 8288.7 |
| 1830 | 313 | 3329.8 | 16072.9 | 8666.2 | 5544.8 |
| 1840 | 162 | 1719.5 | 8300.0 | 4475.2 | 2863.3 |
| 1850 | 152 | 1613.1 | 7786.1 | 4198.1 | 2686.0 |
| 1860 | 151 | 1606.7 | 7755.3 | 4181.5 | 2675.4 |
| 1870 | 136 | 1451.1 | 7004.5 | 3776.7 | 2416.4 |
| 1880 | 118 | 1259.5 | 6079.4 | 3277.9 | 2097.3 |
| 1890 | 117 | 1242.4 | 5997.2 | 3233.6 | 2068.9 |
| 1900 | 116 | 1237.1 | 5971.5 | 3219.7 | 2060.0 |

Biomass was the product of abundance (N) and average weight at age (W). We assumed weight as being constant throughout the time series.

Tables L and M show the output values of trophic level decomposition for each model. Used to generate Table 4 (The difference in trophic level decomposition (*sensu* Lindeman) between the CAB and RAB models).

**Table L. Trophic level decomposition contemporary alosine biomass (CAB) model in absolute values (t/km^2^/year).**

| **Node CAB** | **Group name/**  **Trophic level** | **I** | **II** | **III** | **IV** | **V** | **VI** | **VII** | **VIII** | **IX** | **X** | **XI** |
| --- | --- | --- | --- | --- | --- | --- | --- | --- | --- | --- | --- | --- |
| 1 | Phytoplankton | 3637 | 0 | 0 | 0 | 0 | 0 | 0 | 0 | 0 | 0 | 0 |
| 2 | Bacteria | 0 | 698.4 | 0 | 0 | 0 | 0 | 0 | 0 | 0 | 0 | 0 |
| 3 | Microzooplankton | 0 | 599 | 167.3 | 0 | 0 | 0 | 0 | 0 | 0 | 0 | 0 |
| 4 | Copepods S | 0 | 913.1 | 66.07 | 18.46 | 0 | 0 | 0 | 0 | 0 | 0 | 0 |
| 5 | Copepods L | 0 | 699.5 | 121.3 | 12.93 | 2.047 | 0 | 0 | 0 | 0 | 0 | 0 |
| 6 | Gelatinous Zooplankton | 0 | 35.32 | 98.81 | 11.17 | 1.816 | 0.102 | 0 | 0 | 0 | 0 | 0 |
| 7 | Micronekton | 0 | 251.4 | 349.1 | 46.54 | 6.694 | 0.616 | 0 | 0 | 0 | 0 | 0 |
| 8 | Macrobenthos polychaete | 0 | 188 | 55.91 | 11.21 | 1.575 | 0.231 | 0.0233 | 0.000274 | 0 | 0 | 0 |
| 9 | Macrobenthos crustaceans | 0 | 65.71 | 45.41 | 10.66 | 1.743 | 0.253 | 0.0313 | 0.00247 | 0 | 0 | 0 |
| 10 | Macrobenthos mollusks | 0 | 87.41 | 25.8 | 2.67 | 0.389 | 0.0579 | 0.0062 | 0.000119 | 0 | 0 | 0 |
| 11 | Macrobenthos others | 0 | 88.39 | 43.78 | 9.041 | 1.455 | 0.216 | 0.026 | 0.00179 | 0 | 0 | 0 |
| 12 | Megabenthos filters | 0 | 44.14 | 5.345 | 0 | 0 | 0 | 0 | 0 | 0 | 0 | 0 |
| 13 | Megabenthos others | 0 | 11.06 | 24.23 | 6.244 | 1.142 | 0.178 | 0.0261 | 0.00303 | 0.000093 | 0 | 0 |
| 14 | Shrimp | 0 | 3.456 | 5.124 | 1.031 | 0.161 | 0.0243 | 0.00282 | 0.000184 | 0 | 0 | 0 |
| 15 | Mesopelagic | 0 | 0.0006 | 0.22 | 0.0468 | 0.0072 | 0.000955 | 0.000073 | 0.000007 | 0 | 0 | 0 |
| 16 | Atlantic herring | 0 | 0 | 16.97 | 9.5 | 1.713 | 0.237 | 0.0231 | 0.00103 | 0 | 0 | 0 |
| 17 | Alosines | 0 | 0 | 0.485 | 0.188 | 0.036 | 0.00464 | 0.000578 | 0.000051 | 0.000004 | 0 | 0 |
| 18 | Atlantic menhaden S | 0 | 14.53 | 8.903 | 1.51 | 0.119 | 0.00861 | 0 | 0 | 0 | 0 | 0 |
| 19 | Atlantic menhaden M | 0 | 9.279 | 9.2 | 1.56 | 0.123 | 0.00889 | 0 | 0 | 0 | 0 | 0 |
| 20 | Atlantic menhaden L | 0 | 0.726 | 1.191 | 0.202 | 0.016 | 0.00115 | 0 | 0 | 0 | 0 | 0 |
| 21 | Anchovies | 0 | 0.397 | 9.68 | 13.18 | 1.759 | 0.253 | 0.0233 | 0 | 0 | 0 | 0 |
| 22 | Mackerel | 0 | 0 | 0.686 | 0.511 | 0.282 | 0.0451 | 0.00637 | 0.000643 | 0.000024 | 0 | 0 |
| 23 | Squid | 0 | 0 | 1.349 | 1.166 | 0.293 | 0.0536 | 0.0083 | 0.00104 | 0.000106 | 0.000004 | 0 |
| 24 | Butterfish | 0 | 0.19 | 0.599 | 0.838 | 0.126 | 0.0207 | 0.00208 | 0.000176 | 0.000015 | 0 | 0 |
| 25 | Small pelagics | 0 | 0.0035 | 0.824 | 0.265 | 0.0591 | 0.00744 | 0.000874 | 0.000073 | 0.000002 | 0 | 0 |
| 26 | Bluefish S | 0 | 0 | 0.146 | 0.308 | 0.306 | 0.0474 | 0.00719 | 0.000784 | 0.000042 | 0.000003 | 0 |
| 27 | Bluefish M | 0 | 0 | 0.0245 | 0.0909 | 0.0773 | 0.0132 | 0.00201 | 0.00023 | 0.000016 | 0.000001 | 0 |
| 28 | Bluefish L | 0 | 0 | 0.0109 | 0.172 | 0.15 | 0.0331 | 0.00562 | 0.000743 | 0.000074 | 0.000006 | 0 |
| 29 | Striped bass S | 0 | 0 | 0.541 | 0.58 | 0.358 | 0.0527 | 0.00774 | 0.000814 | 0.000036 | 0 | 0 |
| 30 | Striped bass M | 0 | 0 | 0.748 | 0.897 | 0.556 | 0.099 | 0.0158 | 0.00197 | 0.000172 | 0.000015 | 0 |
| 31 | Striped bass L | 0 | 0 | 0.197 | 0.398 | 0.27 | 0.0467 | 0.00715 | 0.000849 | 0.000065 | 0.000004 | 0 |
| 32 | Weakfish S | 0 | 0 | 0.561 | 0.949 | 0.507 | 0.0694 | 0.00946 | 0.000806 | 0.000004 | 0 | 0 |
| 33 | Weakfish M | 0 | 0 | 0.239 | 0.552 | 0.417 | 0.0603 | 0.00859 | 0.000832 | 0.000023 | 0 | 0 |
| 34 | Weakfish L | 0 | 0 | 0.0144 | 0.0372 | 0.0302 | 0.00552 | 0.000821 | 0.000094 | 0.000005 | 0 | 0 |
| 35 | Dogfish S | 0 | 0 | 0.181 | 0.36 | 0.121 | 0.0274 | 0.00424 | 0.000549 | 0.000058 | 0.000005 | 0 |
| 36 | Dogfish L | 0 | 0 | 0.439 | 0.756 | 0.35 | 0.0761 | 0.0122 | 0.00156 | 0.000154 | 0.000012 | 0 |
| 37 | Atlantic cod S | 0 | 0 | 0.0932 | 0.0726 | 0.015 | 0.00243 | 0.00034 | 0.000038 | 0.000003 | 0 | 0 |
| 38 | Atlantic cod M | 0 | 0 | 0.103 | 0.128 | 0.0499 | 0.0095 | 0.0015 | 0.000188 | 0.000017 | 0.000001 | 0 |
| 39 | Atlantic cod L | 0 | 0 | 0.0385 | 0.0943 | 0.046 | 0.00994 | 0.0016 | 0.000208 | 0.00002 | 0.000002 | 0 |
| 40 | Haddock | 0 | 0 | 0.882 | 0.702 | 0.185 | 0.0344 | 0.00497 | 0.000574 | 0.000046 | 0 | 0 |
| 41 | Hake | 0 | 0 | 1.144 | 1.679 | 0.297 | 0.0566 | 0.00727 | 0.000646 | 0.000065 | 0 | 0 |
| 42 | Croaker | 0 | 0 | 0.42 | 0.232 | 0.07 | 0.0135 | 0.00229 | 0.000307 | 0.000032 | 0.000001 | 0 |
| 43 | Yellowtail flounder S | 0 | 0 | 0.103 | 0.0721 | 0.0153 | 0.00248 | 0.00035 | 0.000041 | 0.000003 | 0 | 0 |
| 44 | Yellowtail flounder L | 0 | 0 | 0.2 | 0.0975 | 0.0213 | 0.0034 | 0.000483 | 0.000055 | 0.000004 | 0 | 0 |
| 45 | Summer flounder S | 0 | 0 | 0.0267 | 0.0605 | 0.0363 | 0.00714 | 0.0012 | 0.00016 | 0.000016 | 0.000001 | 0 |
| 46 | Summer flounder L | 0 | 0 | 0.0365 | 0.237 | 0.185 | 0.0423 | 0.00707 | 0.000963 | 0.000102 | 0.000009 | 0 |
| 47 | Skate | 0 | 0 | 1.678 | 1.587 | 0.569 | 0.116 | 0.0187 | 0.00254 | 0.000267 | 0.000017 | 0 |
| 48 | Demersal benthivores | 0 | 0 | 1.005 | 0.64 | 0.18 | 0.0289 | 0.00431 | 0.000518 | 0.000041 | 0 | 0 |
| 49 | Demersal piscivores | 0 | 0 | 0.166 | 0.322 | 0.138 | 0.0311 | 0.00525 | 0.000741 | 0.000084 | 0.000007 | 0 |
| 50 | Demersal omnivores | 0 | 0 | 0.395 | 0.596 | 0.185 | 0.0382 | 0.00608 | 0.000874 | 0.000098 | 0.000006 | 0 |
| 51 | Medium pelagic | 0 | 0 | 0.0156 | 0.103 | 0.0823 | 0.0175 | 0.0031 | 0.000419 | 0.000045 | 0.000004 | 0 |
| 52 | Coastal sharks | 0 | 0 | 0.00224 | 0.0099 | 0.00751 | 0.00217 | 0.000414 | 0.000062 | 0.000007 | 0.000001 | 0 |
| 53 | Pelagic sharks | 0 | 0.0006 | 0.0007 | 0.0039 | 0.0039 | 0.0014 | 0.00027 | 0.000041 | 0.000005 | 0 | 0 |
| 54 | Large pelagics (HMS) | 0 | 0 | 0.0713 | 0.23 | 0.147 | 0.024 | 0.00345 | 0.000362 | 0.000019 | 0.000001 | 0 |
| 55 | Pinnipeds | 0 | 0 | 0.0193 | 0.0888 | 0.0651 | 0.016 | 0.00281 | 0.00039 | 0.000044 | 0.000004 | 0 |
| 56 | Baleen whales | 0 | 0.0192 | 0.923 | 0.445 | 0.087 | 0.0165 | 0.00199 | 0.000194 | 0.000017 | 0 | 0 |
| 57 | Odontocetes | 0 | 0 | 0.0815 | 0.398 | 0.297 | 0.0679 | 0.012 | 0.00167 | 0.000188 | 0.000019 | 0 |
| 58 | Seabirds | 0 | 0 | 0.0119 | 0.0309 | 0.018 | 0.00379 | 0.000611 | 0.000077 | 0.000007 | 0.000001 | 0 |
| 59 | Detritus | 3079 | 0 | 0 | 0 | 0 | 0 | 0 | 0 | 0 | 0 | 0 |
|  | Total | 6717 | 3710 | 1069 | 171.8 | 27.4 | 3.394 | 0.347 | 0.0312 | 0.00202 | 0.000123 | 0 |

Trophic levels *sensu Lindeman* [35]

**Table M. Trophic level decomposition restored alosine biomass (RAB) model in absolute values (t/km^2^/year).**

| **Node  RAB** | **Group name / Trophic level** | **I** | **II** | **III** | **IV** | **V** | **VI** | **VII** | **VIII** | **IX** | **X** | **XI** |
| --- | --- | --- | --- | --- | --- | --- | --- | --- | --- | --- | --- | --- |
| 1 | Phytoplankton | 3637 | 0 | 0 | 0 | 0 | 0 | 0 | 0 | 0 | 0 | 0 |
| 2 | Bacteria | 0 | 698.4 | 0 | 0 | 0 | 0 | 0 | 0 | 0 | 0 | 0 |
| 3 | Microzooplankton | 0 | 599 | 167.3 | 0 | 0 | 0 | 0 | 0 | 0 | 0 | 0 |
| 4 | Copepods S | 0 | 913.1 | 66.07 | 18.46 | 0 | 0 | 0 | 0 | 0 | 0 | 0 |
| 5 | Copepods L | 0 | 699.5 | 121.3 | 12.93 | 2.047 | 0 | 0 | 0 | 0 | 0 | 0 |
| 6 | Gelatinous Zooplankton | 0 | 35.32 | 98.81 | 11.17 | 1.814 | 0.102 | 0 | 0 | 0 | 0 | 0 |
| 7 | Micronekton | 0 | 316.9 | 289.2 | 42.2 | 5.483 | 0.616 | 0 | 0 | 0 | 0 | 0 |
| 8 | Macrobenthos polychaete | 0 | 191 | 58.74 | 9.662 | 1.447 | 0.193 | 0.0225 | 0 | 0 | 0 | 0 |
| 9 | Macrobenthos crustaceans | 0 | 75.52 | 45.76 | 9.331 | 1.386 | 0.196 | 0.0234 | 0.00171 | 0 | 0 | 0 |
| 10 | Macrobenthos mollusks | 0 | 87.41 | 26.2 | 2.311 | 0.351 | 0.0478 | 0.0057 | 0 | 0 | 0 | 0 |
| 11 | Macrobenthos others | 0 | 93.22 | 47.41 | 8.61 | 1.341 | 0.189 | 0.0236 | 0.00157 | 0 | 0 | 0 |
| 12 | Megabenthos filters | 0 | 44.14 | 5.345 | 0 | 0 | 0 | 0 | 0 | 0 | 0 | 0 |
| 13 | Megabenthos others | 0 | 13.41 | 23.34 | 5.144 | 0.839 | 0.127 | 0.0176 | 0.00211 | 0.00006 | 0 | 0 |
| 14 | Shrimp | 0 | 5.31 | 8.085 | 1.405 | 0.223 | 0.0313 | 0.00389 | 0.000226 | 0 | 0 | 0 |
| 15 | Mesopelagic | 0 | 0.0011 | 0.401 | 0.0823 | 0.0121 | 0.00154 | 0.000106 | 0.00001 | 0 | 0 | 0 |
| 16 | Atlantic herring | 0 | 0 | 30.94 | 13.97 | 2.245 | 0.299 | 0.0291 | 0.000988 | 0 | 0 | 0 |
| 17 | Alosines | 0 | 0 | 4.242 | 1.417 | 0.266 | 0.0304 | 0.0039 | 0.000305 | 0.000012 | 0 | 0 |
| 18 | Atlantic menhaden S | 0 | 18.45 | 11.3 | 1.917 | 0.152 | 0.0109 | 0 | 0 | 0 | 0 | 0 |
| 19 | Atlantic menhaden M | 0 | 10.93 | 10.83 | 1.838 | 0.145 | 0.0105 | 0 | 0 | 0 | 0 | 0 |
| 20 | Atlantic menhaden L | 0 | 1.244 | 2.039 | 0.346 | 0.0273 | 0.00197 | 0 | 0 | 0 | 0 | 0 |
| 21 | Anchovies | 0 | 12.1 | 14.05 | 7.935 | 1.21 | 0.151 | 0.0174 | 0.000296 | 0 | 0 | 0 |
| 22 | Mackerel | 0 | 0 | 1.145 | 0.787 | 0.272 | 0.0445 | 0.00578 | 0.000631 | 0.000026 | 0 | 0 |
| 23 | Squid | 0 | 0 | 2.918 | 2.106 | 0.505 | 0.0842 | 0.0127 | 0.00148 | 0.000142 | 0 | 0 |
| 24 | Butterfish | 0 | 0.19 | 0.637 | 0.813 | 0.116 | 0.0183 | 0.00174 | 0.000128 | 0.000009 | 0 | 0 |
| 25 | Small pelagics | 0 | 0.00828 | 2.043 | 0.574 | 0.111 | 0.0129 | 0.00146 | 0.000113 | 0.000003 | 0 | 0 |
| 26 | Bluefish S | 0 | 0 | 0.354 | 0.395 | 0.197 | 0.034 | 0.00475 | 0.0006 | 0.000043 | 0.000002 | 0 |
| 27 | Bluefish M | 0 | 0 | 0.0527 | 0.1 | 0.0531 | 0.00927 | 0.00133 | 0.000162 | 0.000012 | 0.000001 | 0 |
| 28 | Bluefish L | 0 | 0 | 0.0229 | 0.192 | 0.13 | 0.0265 | 0.00423 | 0.000555 | 0.000053 | 0.000004 | 0 |
| 29 | Striped bass S | 0 | 0 | 0.848 | 0.653 | 0.23 | 0.0362 | 0.00489 | 0.000582 | 0.000034 | 0 | 0 |
| 30 | Striped bass M | 0 | 0 | 0.975 | 0.918 | 0.374 | 0.0684 | 0.0103 | 0.00136 | 0.00013 | 0.000009 | 0 |
| 31 | Striped bass L | 0 | 0 | 0.294 | 0.42 | 0.183 | 0.0319 | 0.0046 | 0.000578 | 0.000049 | 0.000002 | 0 |
| 32 | Weakfish S | 0 | 0 | 1.266 | 1.18 | 0.359 | 0.0529 | 0.00652 | 0.000607 | 0.000014 | 0 | 0 |
| 33 | Weakfish M | 0 | 0 | 0.494 | 0.533 | 0.212 | 0.0335 | 0.00433 | 0.000479 | 0.000022 | 0 | 0 |
| 34 | Weakfish L | 0 | 0 | 0.0283 | 0.0392 | 0.0169 | 0.00318 | 0.00045 | 0.000056 | 0.000004 | 0 | 0 |
| 35 | Dogfish S | 0 | 0 | 0.269 | 0.525 | 0.161 | 0.0326 | 0.0047 | 0.000585 | 0.000059 | 0.000004 | 0 |
| 36 | Dogfish L | 0 | 0 | 0.455 | 0.794 | 0.315 | 0.0617 | 0.00931 | 0.00116 | 0.000108 | 0.000007 | 0 |
| 37 | Atlantic cod S | 0 | 0 | 0.319 | 0.219 | 0.0408 | 0.00615 | 0.000841 | 0.000092 | 0.000006 | 0 | 0 |
| 38 | Atlantic cod M | 0 | 0 | 0.189 | 0.231 | 0.0775 | 0.0136 | 0.00202 | 0.00025 | 0.000021 | 0.000001 | 0 |
| 39 | Atlantic cod L | 0 | 0 | 0.0832 | 0.207 | 0.0869 | 0.0165 | 0.00251 | 0.000317 | 0.00003 | 0.000002 | 0 |
| 40 | Haddock | 0 | 0 | 0.93 | 0.685 | 0.163 | 0.0271 | 0.00381 | 0.000426 | 0.000031 | 0 | 0 |
| 41 | Hake | 0 | 0 | 2.111 | 2.156 | 0.407 | 0.0674 | 0.00905 | 0.000688 | 0.000064 | 0 | 0 |
| 42 | Croaker | 0 | 0 | 0.443 | 0.227 | 0.0557 | 0.0107 | 0.00171 | 0.000229 | 0.000023 | 0.000001 | 0 |
| 43 | Yellowtail flounder S | 0 | 0 | 0.11 | 0.0681 | 0.0129 | 0.00194 | 0.000266 | 0.00003 | 0.000002 | 0 | 0 |
| 44 | Yellowtail flounder L | 0 | 0 | 0.206 | 0.0956 | 0.018 | 0.00274 | 0.000371 | 0.000043 | 0.000002 | 0 | 0 |
| 45 | Summer flounder S | 0 | 0 | 0.106 | 0.172 | 0.0823 | 0.0162 | 0.00256 | 0.000345 | 0.000035 | 0.000003 | 0 |
| 46 | Summer flounder L | 0 | 0 | 0.142 | 0.57 | 0.349 | 0.0729 | 0.0116 | 0.00157 | 0.000164 | 0.000013 | 0 |
| 47 | Skate | 0 | 0 | 1.797 | 1.586 | 0.485 | 0.0891 | 0.0137 | 0.0018 | 0.000185 | 0.000006 | 0 |
| 48 | Demersal benthivores | 0 | 0 | 1.369 | 0.79 | 0.17 | 0.0269 | 0.00381 | 0.000469 | 0.000035 | 0 | 0 |
| 49 | Demersal piscivores | 0 | 0 | 0.29 | 0.507 | 0.193 | 0.0388 | 0.00622 | 0.000846 | 0.000093 | 0.000007 | 0 |
| 50 | Demersal omnivores | 0 | 0 | 0.824 | 1.103 | 0.306 | 0.0572 | 0.00887 | 0.00123 | 0.00014 | 0.000005 | 0 |
| 51 | Medium pelagic | 0 | 0 | 0.0188 | 0.112 | 0.0743 | 0.0149 | 0.00243 | 0.000323 | 0.000032 | 0.000003 | 0 |
| 52 | Coastal sharks | 0 | 0 | 0.00315 | 0.011 | 0.00698 | 0.00183 | 0.000324 | 0.000047 | 0.000005 | 0.000001 | 0 |
| 53 | Pelagic sharks | 0 | 0.00171 | 0.00254 | 0.0124 | 0.0108 | 0.00326 | 0.00059 | 0.000086 | 0.000011 | 0.000001 | 0 |
| 54 | Large pelagics (HMS) | 0 | 0 | 0.12 | 0.242 | 0.0985 | 0.0164 | 0.00217 | 0.00024 | 0.000014 | 0 | 0 |
| 55 | Pinnipeds | 0 | 0 | 0.0453 | 0.156 | 0.0967 | 0.0216 | 0.00361 | 0.000487 | 0.000054 | 0.000005 | 0 |
| 56 | Baleen whales | 0 | 0.0192 | 0.973 | 0.405 | 0.0806 | 0.0132 | 0.00159 | 0.000135 | 0.00001 | 0 | 0 |
| 57 | Odontocetes | 0 | 0 | 0.871 | 3.389 | 1.873 | 0.405 | 0.0653 | 0.00884 | 0.000947 | 0.000091 | 0 |
| 58 | Seabirds | 0 | 0 | 0.0197 | 0.0579 | 0.0287 | 0.0057 | 0.000873 | 0.00011 | 0.00001 | 0.000001 | 0 |
| 59 | Detritus | 3125 | 0 | 0 | 0 | 0 | 0 | 0 | 0 | 0 | 0 | 0 |
|  | Total | 6762 | 3815 | 1054 | 171.8 | 26.94 | 3.486 | 0.368 | 0.0349 | 0.00269 | 0.00017 | 0 |

Trophic levels *sensu Lindeman* [35]

**Diet matrices**

The Contemporary Alosine Biomass (CAB) model data source can be found above in the nodes’ documentation description. For Restored Alosine Biomass diets were modified during the balancing process, allowing more predation on anadromous alosine group (S1 and S12 Tables).

References

1. Link J, Griswold C, Methratta T, Gunnrd J. Documentation for the Energy Modeling and Analysis Exercise (EMAX) [Internet]. Northeast Fisheries Science Center Reference Document. Woods Hole, MA; 2006. Available: http://www.nefsc.noaa.gov/publications/crd/crd0615/crd0615.pdf

2. Link J, Overholtz W, O’Reilly J, Green J, Dow D, Palka D, et al. The Northeast U.S. continental shelf Energy Modeling and Analysis exercise (EMAX): Ecological network model development and basic ecosystem metrics. J Mar Syst. Elsevier B.V.; 2008;74: 453–474. doi:10.1016/j.jmarsys.2008.03.007

3. Buchheister A, Miller TJ, Houde ED, Loewensteiner DA. Technical Documentation of the Northwest Atlantic Continental Shelf (NWACS) Ecosystem Model [Internet]. Washington, D.C.; 2017. Available: http://hjort.cbl.umces.edu/NWACS/TS_694_17_NWACS_Model_Documentation.pdf

4. Buchheister A, Miller TJ, Houde ED. Evaluating Ecosystem-Based Reference Points for Atlantic Menhaden. Mar Coast Fish. Taylor & Francis; 2017;9: 457–478. doi:10.1080/19425120.2017.1360420

5. Link JS. Adding rigor to ecological network models by evaluating a set of pre-balance diagnostics: A plea for PREBAL. Ecol Modell. 2010;221: 1580–1591. doi:10.1016/j.ecolmodel.2010.03.012

6. Aydin KY, Gaichas S, Ortiz I, Kinzey D, Friday N. A comparison of the Bering Sea, Gulf of Alaska, and Aleutian Islands large marine ecosystems throug food web modeling [Internet]. NOAA Technical Memorandum NMFS-AFSC. no. 178. 2007 Feb. Available: www.afsc.noaa.gov

7. Kearney KA. An analysis of marine ecosystem dynamics through development of a coupled physical-biogeochemical-fisheries food web model [Internet]. Princenton University. 2012. Available: https://search.proquest.com/docview/1240669687?accountid=14572

8. Kearney KA. ecopath_matlab: A Matlab-based implementation of the Ecopath food web algorithm. J Open Source Softw. 2017;2. doi:10.21105/joss.00064

9. Christensen V, Walters CJ, Pauly D, Forrest R. Ecopath with Ecosim version 6 User Guide. Fish Centre, Univ Br Columbia, Vancouver, Canada. 2008;281: 1–235. Available: http://sources.ecopath.org/trac/Ecopath/wiki/UsersGuide

10. Heymans JJ, Coll M, Link JS, Mackinson S, Steenbeek J, Walters C, et al. Best practice in Ecopath with Ecosim food-web models for ecosystem-based management. Ecological Modelling. 2016: 173–184. doi:10.1016/j.ecolmodel.2015.12.007

11. Overholtz WJ, Friedland KD. Recovery of the Gulf of Maine – Georges Bank Atlantic herring (Clupea harengus) complex: perspectives based on bottom trawl survey data. Fish Bull. 1998;100: 593–608.

12. NEFSC. 54th Northeast Regional Stock Assessment Workshop (54th SAW) Assessment Report. [Internet]. US Dept Commer, Northeast Fish Sci Cent Ref Doc. National Marine Fisheries Service, 166 Water Street, Woods Hole, MA 02543-1026; 2012. Available: http://www.nefsc.noaa.gov/nefsc/ publications/

13. Christensen V, Beattie A, Buchanan C, Ma H, Martell SJD, Latour RJ, et al. Fisheries ecosystem model of the Chesapeake Bay: Methodology, parameterization, and model exploration. NOAA Tech Memo. 2009;

14. SEDAR. SEDAR 40 - Atlantic Menhaden Stock Assessment Report [Internet]. SEDAR, North Charleston, SC; 2015. Available: http://www.sefsc.noaa.gov/sedar/Sedar_Workshops.jsp?WorkshopNum=40

15. Gascuel D, Morissette L, Palomares MLD, Christensen V. Trophic flow kinetics in marine ecosystems: Toward a theoretical approach to ecosystem functioning. Ecol Modell. 2008;217: 33–47. doi:10.1016/j.ecolmodel.2008.05.012

16. Palomares MLD, Pauly D. Predicting food consumption of fish populations as functions of mortality, food type, morphometrics, temperature and salinity. Mar Freshw Res. 1998;49: 447. doi:10.1071/MF98015

17. Salerno DJ, Burnett J, Ibara RM. Age, Growth, Maturity, and Spatial Distribution of Bluefish, Pomatomus saltatrix (Linnaeus), off the Northeast Coast of the United States, 1985-96. J Northw Atl Fish Sci. 2001;29: 31–39. Available: http://journal.nafo.int

18. Terceiro M, Ross JL. A comparison of alternative methods for the estimation of age from length data for Atlantic coast bluefish ( Pomatomus saltatrixj. Fish Bull. 1993;91: 534–549. Available: https://www.st.nmfs.noaa.gov/spo/FishBull/913/terceiro.pdf

19. Froese R, Pauly D. FishBase. In: World Wide Web electronic publication. [Internet]. 2018. Available: www.fishbase.org, ( 06/2018 )

20. Anonymous. Levitus map. [Internet]. 2001. Available: http://dpo.ori.ou-tokyo.ac.jp/ocean/toolmap/Levitus-map.html

21. Shephard G, Grimes CB. Geographic and historic variations in growth of weakfish, Cynoscion regalis, in the middle Atlantic bight. Fish Bull. 1983;81: 803–813. Available: https://www.st.nmfs.noaa.gov/spo/FishBull/81-4/shepherd.pdf

22. Bubley WJ, Kneebone J, Sulikowski JA, Tsang PCW. Reassessment of spiny dogfish Squalus acanthias age and growth using vertebrae and dorsal-fin spines. J Fish Biol. Wiley/Blackwell (10.1111); 2012;80: 1300–1319. doi:10.1111/j.1095-8649.2011.03171.x

23. NEFSC. 55th Northeast Regional Stock Assessment Workshop (55th SAW) Assessment Report [Internet]. US Dept Commer, Northeast Fish Sci Cent Ref Doc. 13-11; 845 p. National Marine Fisheries Service, 166 Water Street, Woods Hole, MA 02543-1026; 2013. Available: http://www.nefsc.noaa.gov/nefsc/ publications/

24. Magnussen E. Interpopulation comparison of growth patterns of 14 fish species on Faroe Bank: are all fishes on the bank fast-growing? J Fish Biol. 2007;71: 453–475. doi:10.1111/j.1095-8649.2007.01502.x

25. Pauly D. On the interrelationships between natural mortality, growth parameters, and mean environmental temperature in 175 fish stocks. ICES J Mar Sci. Oxford University Press; 1980;39: 175–192. doi:10.1093/icesjms/39.2.175

26. Smith RW, Daiber FC. Biology of the summer flounder, Paralichthys dentatus, in Delaware Bay. Fish Bull. 1977;75: 823–830.

27. NEFSC. 58th Northeast Regional Stock Assessment Workshop (58th SAW) Assessment Report. [Internet]. US Dept Commer, Northeast Fish Sci Cent Ref Doc. National Marine Fisheries Service, 166 Water Street, Woods Hole, MA 02543-1026; 2014. Available: http://www.nefsc.noaa.gov/ publications/

28. NEFSC. 57th Northeast Regional Stock Assessment Workshop (57th SAW) Assessment Report. US Dept Commer, Northeast Fish Sci Cent Ref Doc 13-16. National Marine Fisheries Service, 166 Water Street, Woods Hole, MA 02543-1026; 2013;13–16: 967 p. Available: http://www.nefsc.noaa.gov/nefsc/ publications/

29. Sagarese SR, Frisk MG, Cerrato RM, Sosebee KA, Musick JA, Rago PJ. Diel Variations in Survey Catch Rates and Survey Catchability of Spiny Dogfish and their Pelagic Prey in the Northeast U.S. Continental Shelf Large Marine Ecosystem. Mar Coast Fish. Taylor & Francis; 2016;8: 244–262. doi:10.1080/19425120.2015.1135219

30. Araújo JN, Bundy a. Description of three Ecopath with Ecosim ecosystem models developed for the Bay of Fundy , Western Scotian Shelf and NAFO Division 4X Bedford Institute of Oceanography Canadian Technical Report of Fisheries and Aquatic Sciences 2952. 2011; 189. doi:10.13140/2.1.2383.9688

31. Pauly D. Food consumption by tropical and temperate marine fishes: some generalizations. J Fish Biol. 1989;35: 11–20. doi:10.1111/j.1095-8649.1989.tb03041.x

32. NEFSC. 59th Northeast Regional Stock Assessment Workshop (59th SAW) Assessment Report. [Internet]. US Dept Commer, Northeast Fish Sci Cent Ref Doc. 14-09; 782 p. 2014. Available: http://www.nefsc.noaa.gov/publications/

33. NEFSC. Assessment or Data Updates of 13 Northeast Groundfish Stocks through 2010. [Internet]. US Dept Commer, Northeast Fish Sci Cent Ref Doc. 12-06; 789 p. 2012. Available: http://www.nefsc.noaa.gov/nefsc/publications/

34. ASMFC. Atlantic States Marine Fisheries Commission Atlantic - Atlantic Croaker 2010 Benchmark Stock Assessment. 2010.

35. Christensen V, Walters CJ. Ecopath with Ecosim: methods, capabilities and limitations. Ecol Modell. 2004;172: 109–139. doi:10.1016/j.ecolmodel.2003.09.003
